# Fibroblast growth factor signaling is required for early somatic gonad development in zebrafish

**DOI:** 10.1371/journal.pgen.1006993

**Published:** 2017-09-05

**Authors:** Dena M. Leerberg, Kaori Sano, Bruce W. Draper

**Affiliations:** 1 Department of Molecular and Cellular Biology, University of California, Davis, California, United States of America; 2 Graduate school of Science and Technologies, Sophia University, Tokyo, Tokyo, Japan; University of Pennsylvania School of Medicine, UNITED STATES

## Abstract

The vertebrate ovary and testis develop from a sexually indifferent gonad. During early development of the organism, primordial germ cells (the gamete lineage) and somatic gonad cells coalesce and begin to undergo growth and morphogenesis to form this bipotential gonad. Although this aspect of development is requisite for a fertile adult, little is known about the genetic regulation of early gonadogenesis in any vertebrate. Here, we provide evidence that fibroblast growth factor (Fgf) signaling is required for the early growth phase of a vertebrate bipotential gonad. Based on mutational analysis in zebrafish, we show that the Fgf ligand 24 (Fgf24) is required for proliferation, differentiation, and morphogenesis of the early somatic gonad, and as a result, most *fgf24* mutants are sterile as adults. Additionally, we describe the ultrastructural elements of the early zebrafish gonad and show that distinct somatic cell populations can be identified soon after the gonad forms. Specifically, we show that *fgf24* is expressed in an epithelial population of early somatic gonad cells that surrounds an inner population of mesenchymal somatic gonad cells that are in direct contact with the germ cells, and that *fgf24* is required for stratification of the somatic tissue. Furthermore, based on gene expression analysis, we find that differentiation of the inner mesenchymal somatic gonad cells into functional cell types in the larval and early juvenile-stage gonad is dependent on Fgf24 signaling. Finally, we argue that the role of Fgf24 in zebrafish is functionally analogous to the role of tetrapod FGF9 in early gonad development.

## Author summary

The genes involved in the early stages of vertebrate gonad development remain largely undefined. The gonad begins to form when primordial germ cells and somatic gonad precursor cells coalesce during early development. However, we know little about the signaling events that lead to the subsequent morphogenesis of the early developing gonadal primordium. Using the zebrafish, a model vertebrate, we show that the early somatic gonad organizes into a bi-layered structure, with an outer epithelial layer surrounding an inner mesenchymal core. We demonstrate that the gene encoding the Fibroblast growth factor 24 ligand, *fgf24*, is expressed by the epithelial population. Utilizing a null mutation in *fgf24*, we show that Fgf24 signaling is required for the expression of several genes by the inner mesenchymal cells, including those encoding transcription factors, a hormone biosynthesis enzyme, and a TGF-β ligand. Furthermore, the somatic cells in *fgf24* mutant gonads have reduced proliferation rates, do not organize into the bi-layered structure seen in wild-type gonads, and most *fgf24* mutants are infertile as adults due to the inability of the somatic gonad to support germ cell development. Finally, we argue that the function of Fgf24 during development of the early teleost gonad is analogous to the proposed role of FGF9 during development of the early tetrapod gonad.

## Introduction

The vertebrate gonad consists of germ cells, the lineage directly responsible for creating the next generation, and somatic gonad cells (SGCs). The somatic gonad serves two important functions. First, it creates an environment that protects germ cells and nurtures their development. For example, with the exception of mammalian females, most animals examined retain the ability to produce gametes throughout their adult life, an activity permitted by the presence of germline stem cells [1]. SGCs form the niche that is required to maintain these germline stem cells. In the adult mouse testis, several SGC types (Sertoli, Leydig, and peritubular myoid cells) secrete growth factors that promote proliferation and suppress differentiation of the germline stem cell population, thereby maintaining fertility through adulthood [2, 3]. Second, a subset of SGCs secretes hormones required for the development of secondary sexual characteristics, so defects in gonad development can result in disorders of sexual development (reviewed in [4]). Although the importance of the somatic gonad to fertility is clear, much remains to be learned about the genes that regulate its early development.

In mammals, the somatic gonad is derived from intermediate and lateral plate mesoderm, and its early development has been divided into three major steps: initiation, growth, and sexual differentiation. During the initiation phase, a portion of coelomic epithelium on the ventral surface of the mesonephros begins to thicken to form the bilateral genital ridges [5–8]. Soon after their formation, primordial germ cells (PGCs) migrate into the ridges [9]. The growth phase is characterized as a period of somatic and germ cell proliferation that results in a larger, multilayered primordium. Finally, sexual differentiation transforms the bipotential gonads into either an ovary or testis. Although the molecular mechanisms that regulate sexual differentiation of the gonad are relatively well understood (reviewed in [10]), less is known about the genetic regulation of the initiation and growth phases. In mice, several transcription factors have been identified that are required for initiation and growth of the primordium: mutations in *Gata4*, Wilms tumor 1(*Wt1*), Steroidogenic factor 1 (*Sf1/Nr5a1*), Lim homeobox protein 9 (*Lhx9*) or the paired-like homeobox gene *Emx2* cause failure to initiate gonad development or undergo early gonad regression [11–17]. However, it is not known if cell signaling is important for these early events.

The Fgf signaling pathway regulates many developmental events in metazoans. The pathway generally consists of secreted ligands that complex with heparan sulfate proteoglycans in the extracellular matrix to bind and activate transmembrane Fgf receptors [18, 19]. One such ligand, Fgf24, is known to play roles in the development of zebrafish posterior mesoderm, forelimb, and pancreas [20–22]. Fgf24 is a member of the FgfD subfamily of Fgf ligands, which, in zebrafish, consists of six members: *fgf8a*, *fgf8b*, *fgf17*, *fgf18a*, *fgf18b*, and *fgf24* [20, 23, 24]. Although this subfamily is conserved in mammals, the *fgf8* and *fgf18* duplications are a result of the teleost-specific whole-genome duplication [24]. Furthermore, while *Fgf24* is present in basal tetrapods and teleosts (coelacanths and spotted gar, respectively), it was lost early in the tetrapod lineage, so is not present in the mammalian genome [23–27].

In this study, we show that the majority of *fgf24* null mutants (*ika*^*hx118*^, hereafter referred to as *fgf24*^*hx118*^; [21]) are sterile as adults, suggesting that it plays a role in either gonad development or maintenance. We show that *fgf24* is first expressed in a subset of SGCs by 8 days post fertilization (dpf)—a period that we argue is analogous to the early growth stage of mammalian gonad development—and that this expression is required for early somatic gonad proliferation and morphogenesis into a bi-layered tissue that in wild-type normally occurs by 10 dpf. Coincident with bi-layer formation, we show that cells expressing *fgf24* are restricted to the epithelial layer on the surface of the gonad. Furthermore, we show evidence that the cells responding to Fgf24 signaling are a mesenchymal population of SGCs that localizes to the interior of the early gonad, and that loss of Fgf24 function leads to a failure of these cells to differentiate into functional cell types. Finally, we argue that the loss of germ cells in *fgf24* mutants is an indirect consequence of defective somatic gonad development.

## Results

### Background: Zebrafish early gonad development and sex determination

**Fig 1:**
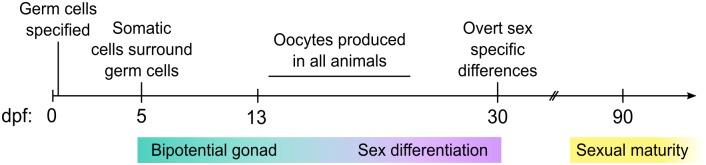
Background: Timeline of zebrafish gonad development. Primordial germ cells are specified shortly after fertilization (mid-blastula, 3 hours post fertilization (hpf)), migrate to their final destination by 24 hpf, and begin associating with somatic cells by 5 dpf. Over the next 1–2 weeks of development, all gonads are bipotential and begin to produce early stage oocytes around 13 dpf. Sex differentiation likely begins prior to 20 dpf but is not overtly apparent until ~30 dpf [36].

Gonads are composed of both germ cells and somatic cells that enclose the germ cells and regulate their development. In vertebrates, the somatic gonad is also the main source of sex hormones that regulate secondary sexual characteristics, such as sexually dimorphic appearances and behavior. Zebrafish primordial germ cells (PGCs) are specified during the early cleavage stage by maternal factors, and shortly thereafter migrate to where the somatic gonad will eventually form [28] (Fig 1). Though it is not known with certainty when formation of the somatic gonad initiates, histological analysis has detected SGCs surrounding germ cells as early as 5 dpf [29].

Domesticated zebrafish lack sex chromosomes and it is still unclear how sex determination is regulated or precisely when it occurs [30]. Zebrafish embryos hatch from the chorion around 3 dpf and are free-swimming larvae by 5 dpf. The transition from the larval to juvenile stage occurs around 25 dpf, which coincides closely to when overt sex-specific differentiation becomes apparent (Fig 1). During the early larval stage (i.e. 3–20 dpf), there are no overt differences between animals that will become male or females, and all animals initially produce varying numbers of early stage oocytes (beginning ~13 dpf; [31]). Mutations that reduce or eliminate germ cells, or more specifically, the ability to produce early stage oocytes during the larval period, result in all male development [32–34]. During this period of development, the somatic gonad is bipotential as evident by the simultaneous expression of genes that will eventually be sex-specific. For example, a subset of somatic cells in the larval gonad expresses the female-specific aromatase-encoding gene, *cyp19a1a*, while neighboring cells express the male-specific *amh* gene ([35]; this report). These studies have led to the hypothesis that oocytes produce a signal that stabilizes female-specific gene expression in the somatic gonad, thereby promoting female development.

### *fgf24* is required for early gonad development

**Fig 2:**
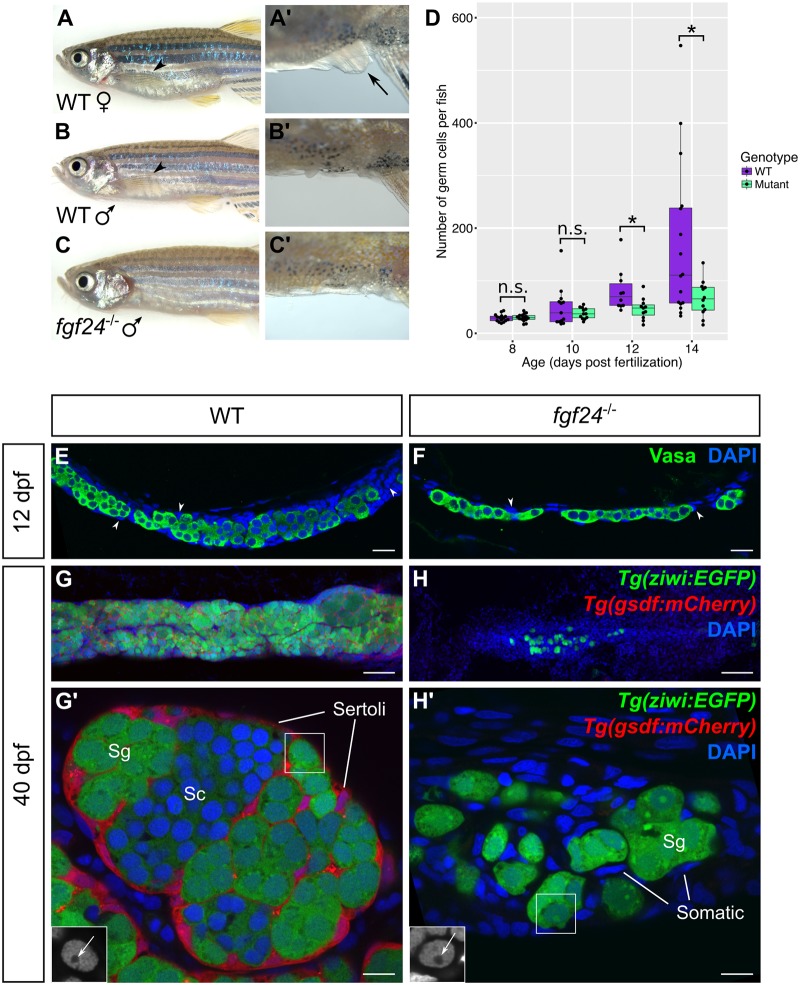
Larval and juvenile *fgf24* mutants have severely underdeveloped gonads and consequently develop as male. (A-C’) *fgf24* mutants are phenotypically male. A wild-type (WT) female has a distended belly (A) and protruding cloaca (arrow, A’) compared to the slender belly and obscured cloaca of the wild-type (B, B’) and mutant (C, C’) males. Note the absence of pectoral fin in the *fgf24* mutant (C) compared to wild-type animals (arrowheads, A, B). (D) Box and whisker plot representing the number of germ cells per wild-type and *fgf24* mutant fish during larval development. Each dot represents the number of germ cells in one animal (Unpaired two-tailed t-test; n = 15 for both 8 dpf wild-type and mutant, n = 13 for 10 dpf wild-type, n = 12 for 10 dpf mutant, n = 10 for 12 dpf wild-type, n = 11 for 12 dpf mutant, n = 16 for 14 dpf wild-type, n = 12 for 14 dpf mutant; * = P < .05; n.s. = no significance). (E-H’) Representative confocal images of 12 dpf (E-F) and 40 dpf (G-H’) wild-type (E, G-G’) and *fgf24* mutant (F, H-H’) gonads. At 12 dpf, gonads of *fgf24* mutants (F) have fewer Vasa positive germ cells (green) compared to wild-type (E). By 40 dpf, wild-type testes have many *Tg(ziwi*:*EGFP)* positive germ cells (green), both premeiotic and spermatogenic, that are enclosed by *Tg(gsdf*:*mCherry)* positive Sertoli cells (red) (G-G’). In contrast, gonads of 40 dpf *fgf24* mutants are unorganized, lack *Tg(gsdf*:*mCherry)* expressing Sertoli cells, and contain few germ cells, all of which are premeiotic (H-H’). G’ and H’ are magnified views G and H, respectively. The boxed nuclei in G’ and H’ are magnified in the respective insets, with an arrow indicating the large nucleoli (DAPI only, in grey). E-H’ are sagittal optical sections with anterior to the left. Nuclei labeled with DAPI (blue). Scale bars = 20 μm for E, F, G, H; 100 μm for G’, H’.

It has previously been shown that zebrafish *fgf24* homozygous mutants, called *fgf24*^*hx118*^, are viable but lack pectoral fins [21]. In addition to this defect, we discovered that all *fgf24*^*hx118*^ mutants were males as adults (Fig 2A–2C’). Previous studies have established that zebrafish lacking germ cells, or the ability to produce early-stage oocytes, invariably develop as phenotypic males [32–34]. We therefore examined 27 *fgf24* mutants at 3.5 months post fertilization (mpf) and found that 21 animals contained no detectable gonads and were thus sterile (~78%). Each of the remaining 6 animals had one small testis measuring approximately 1/3 the size of a wild-type testis (~22%; S1A and S1B Fig). High-resolution confocal analysis of these latter testes revealed an overall wild-type organization where germ and somatic cells were properly arranged into tubule structures that contained germ cells in all stages of spermatogenesis, including mature sperm (S1C and S1D Fig). Furthermore, *in situ* hybridization with the Sertoli cell gene markers *anti-müllerian hormone* (*amh*) and *gonadal soma derived factor* (*gsdf*) showed a similar pattern of expression in both *fgf24* mutants and wild-type controls (S1E–S1H Fig). Finally, while mutant fish that have gonads were unable to induce wild-type females to spawn due to their lack of pectoral fins [37], sperm extracted from these gonads and used for *in vitro* fertilization of wild-type eggs produced viable embryos at frequencies indistinguishable from wild-type sperm (S1 Table).

Although the *fgf24*^*hx118*^ allele is an *N*-ethyl-*N*-nitrosourea (ENU)-induced point mutation that introduces a premature stop codon within exon 4 that should truncate the protein within the core Fgf homology domain [21], the incomplete penetrance of its adult sterility phenotype prompted us to investigate whether this allele was hypomorphic (S1I Fig). To test this, we used the CRISPR/Cas9 genome editing technology to induce a new mutation within exon 3 and identified a 5 bp insertion allele, called *fgf24*^*uc47*^, that results in a translational frameshift, and is therefore expected to be a null mutation (S1I Fig). We found that both *fgf24*^*uc47*^ homozygous mutants and *fgf24*^*hx118/uc47*^ transheterozygotes have a phenotype that is indistinguishable from that caused by the *fgf24*^*hx118*^ mutation alone: the resulting animals are homozygous viable, lack pectoral fins, and develop as male with a partially penetrant sterility defect. In subsequent experiments, we use these two alleles interchangeably and conclude that null mutations in *fgf24* lead to an incompletely penetrant adult sterility defect.

*fgf24* mutants are all male as adults, a phenotype that suggest that mutants may have defects in early gonad development (see Background, above). We therefore first determined if they had defects in early germ cell development by comparing the number of germ cells present in the gonads of wild-type and mutant fish at several stages throughout larval development. For these experiments, we collected confocal images at 5 μm intervals through the whole gonad and quantified distinct germ cells identified by anti-Vasa antibody and DAPI DNA staining. We found that *fgf24* mutants had similar numbers of germ cells to wild-type siblings at 8 and 10 dpf. However, at 12 and 14 dpf, mutant animals had significantly fewer germ cells than their wild-type siblings (Fig 2D–2F).

In addition to our finding that *fgf24* mutants have fewer germ cells than wild-type, they also appear to have fewer SGCs (arrowheads in Fig 2E and 2F). This feature was most apparent when we compared testes isolated from wild-type and *fgf24* mutant animals. A 40 dpf wild-type testis is organized into tubules that contain many premeiotic and spermatogenic germ cells surrounded by SGCs (Fig 2G and 2G’). One such SGC is the Sertoli cell, which expresses the teleost gonad-specific Tgf-β ligand, Gsdf [38]. At 40 dpf, Gsdf-expressing Sertoli cells are abundant in wild-type testes and can be visualized using the *Tg(gsdf*:*mCherry)*^*uc46*^ transgene as previously reported [38] (n = 7; Fig 2G and 2G’). In stark contrast, the gonads of all *fgf24* mutants contain few germ cells that are not organized into tubule-like structures and lack detectable *Tg(gsdf*:*mCherry)*^*uc46*^ expressing SGCs (n = 7; Fig 2H and 2H’). Notably, *gsdf* is also expressed in ovarian granulosa cells (S2 Fig). Thus the inability of *fgf24* mutant gonads to express *Tg(gsdf*:*mCherry)*^*uc46*^ argues that *fgf24* is required for the development of both male and female SGCs. Finally, the testes and ovaries of wild-type juvenile animals (25-90dpf) contain both premeiotic and postmeiotic germ cells at different stages of gametogenesis (Fig 2G’; S2 Fig) in contrast to juvenile *fgf24* mutant gonad, which contain only premeiotic germ cells as evident by their large size and prominent nucleoli (Fig 2H’, inset). In conclusion, loss of *fgf24* function affects the development of both testes and ovaries, which, together with the expression of *fgf24* during the early bipotential phase, suggests that it is required for development of the early bipotential gonad (Fig 1).

### *fgf24* is expressed in somatic cells on the surface of the gonad

**Fig 3:**
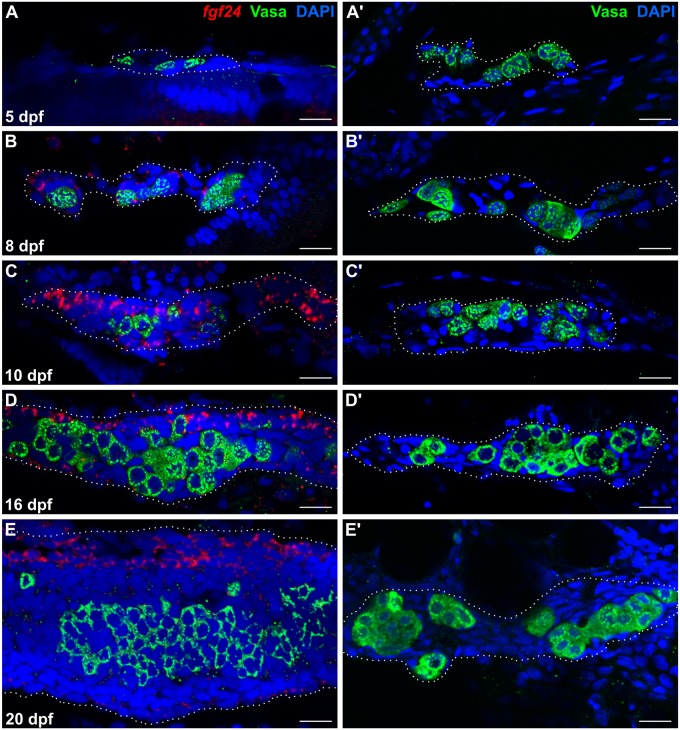
*fgf24* is expressed in somatic gonad cells during wild-type larval development. (A, B, C, D, E) Single plane confocal micrographs of whole-mount larval gonads after fluorescent *in situ* hybridization detecting *fgf24* (red). At 5 dpf, *fgf24* is not detected by fluorescent *in situ* hybridization (A). By 8 dpf, *fgf24* is detected in SGCs (B) and is restricted to an outer layer of SGCs at 10 (C) and 16 (D) dpf. By 20 dpf, expression is primarily restricted to SGCs on the dorsal edge of the gonad (E). (A’, B’, C’, D’, E’) Gonads dissected from *fgf24* mutants age-matched to the wild-type animals depicted in A, B, C, D, and E. Gonads are outlined with white dotted lines. A-E’ are sagittal optical sections where anterior is to the left and dorsal is up. Nuclei labeled with DAPI (blue), germ cells labeled with Vasa (green). Scale bars = 20 μm.

To further test the role of Fgf24 in early bipotential gonad development, we used high resolution fluorescent RNA *in situ* hybridization to determine which gonadal cells express *fgf24*. The larval gonad is a rod-like structure that is oriented along the anterior/posterior axis. Germ cells are restricted to the interior core of the gonad and are surrounded by SGCs (Fig 2E). Because the germ cell phenotype of *fgf24* mutants is first evident by 12 dpf, we examined the expression of *fgf24* mRNA in gonads at various stages between 5 and 20 dpf in whole-mount gonads. In addition, we co-stained gonads for the germ cell-specific Vasa protein to aid in gonad identification during dissection. At 5 dpf, when germ cells and SGCs have just begun to coalesce [29], *fgf24* was not detected (n = 4; Fig 3A). However, by 8 dpf, we could detect *fgf24* expression in some, but not all, SGCs in 23/27 wild-type animals (Fig 3B). In 10 and 16 dpf animals, *fgf24* could be detected in all gonads examined (n = 19 and 12, respectively). As in 8 dpf gonads, *fgf24* was detected exclusively in SGCs, but only in a subset of SGCs that appeared to be restricted to the surface of the gonad (Fig 3C and 3D). Finally, in 20 dpf animals, we continued to detect *fgf24* only in SGCs, though expression appeared highest in a population of cells that localize to the dorsal edge of the gonad (n = 9; Fig 3E). We conclude that *fgf24* is expressed in gonads during the time when the development of *fgf24* mutant gonads begins to deviate from those of wild-type animals (Fig 3A’, 3B’, 3C’, 3D’ and 3E’). Furthermore, these results reveal that there are at least two distinct SGC populations in the larval gonad soon after its formation: *fgf24*(+) and *fgf24*(-).

### Fgf24 signals to an inner layer of somatic gonad cells

**Fig 4:**
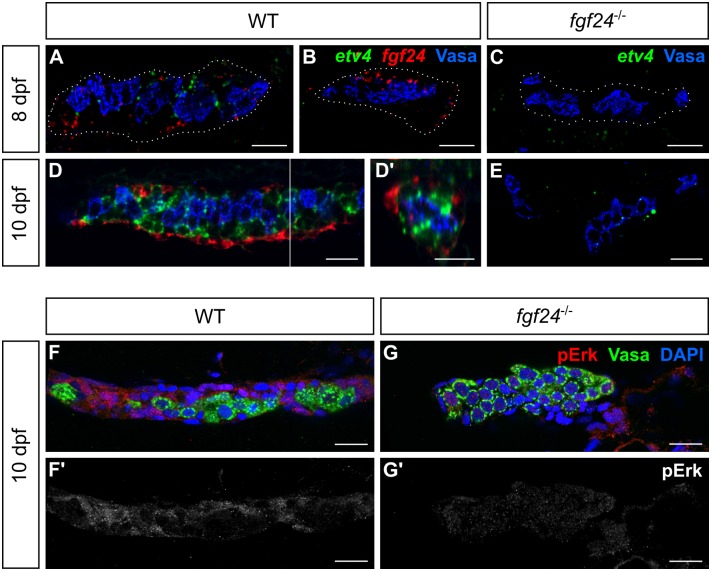
A second, inner layer of somatic gonad cells responds to Fgf24 signaling. (A-D, E) Single plane view of whole mount gonads after fluorescent *in situ* hybridization. (A-B) At 8 dpf, ~85% of wild-type (WT) fish express *fgf24* (red) in SGCs, whereas ~56% express *etv4* (green) (A). (C) *etv4* is undetected in *fgf24* mutant gonads (n = 14). (D) By 10 dpf, SGCs located externally express *fgf24*, whereas an inner layer of SGCs expresses *etv4*. (D’) An orthogonal view of a Z stack reconstruction of the gonad in D (white line). (E) In 10 dpf *fgf24* mutant gonads, *etv4* is expressed at low levels or not at all. Germ cells labeled with Vasa (blue). Gonads are outlined with white dotted lines in A-C. (F-G) Antibody staining of 10 dpf gonads detecting phosphorylated Erk (pErk, red). Nuclei labeled with DAPI (blue), germ cells labeled with Vasa (green). SGCs of wild-type gonads (F), but not *fgf24* mutant gonads (G), show Erk phosphorylation. (F’, G’) are pErk channel only of (F) and (G), respectively. A-D, E-G’ are sagittal optical sections with anterior to the left. Scale bars = 20 μm.

The apparent defect in both the somatic and germ cell components of the gonad led us to investigate which cell type(s) responds to Fgf24 signaling. Fgf signaling can activate downstream signaling cascades that can result in gene expression changes. Transcription of the Ets variant (Etv) family of transcription factors is known to be upregulated by Fgf signaling in many developmental contexts (e.g. [39–41]). We therefore asked whether one of these family members, *etv4*/*pea3*, is expressed in the wild-type larval gonad. At 8 dpf, when we first detect *fgf24* expression in ~85% of wild-type animals (Fig 3B), we can detect expression of *etv4* in SGCs in 56% of wild-type gonads (13/23; Fig 4A and 4B). Notably, it is only detected in gonads that also express *fgf24* (Fig 4A) and is not detected in 8 dpf *fgf24* mutant gonads (n = 14; Fig 4C). By 10 dpf, however, we found that *etv4* is strongly expressed in SGCs in all wild-type gonads examined (n = 25). Interestingly, double *in situ* hybridization of both *etv4* and *fgf24* reveals that *etv4* is expressed in a population of SGCs distinct from that of *fgf24* and one that subtends the *fgf24*-expressing layer (n = 10; Fig 4D and 4D’). In contrast, the gonads of 10 dpf *fgf24* mutant siblings express no, or greatly reduced levels of, *etv4* (n = 8; Fig 4E). These results suggest that Fgf24 acts as a paracrine signal to regulate the development of an inner population of SGCs.

In various contexts, the Fgf activation of *etv4* transcription is mediated by Erk, a terminal kinase of the Map kinase signaling pathway [42, 43]. Erk phosphorylation by the upstream kinase, Mek, allows it to translocate to the nucleus and activate numerous transcription factors (reviewed in [44]). We therefore asked whether Erk is phosphorylated in larval SGCs. Indeed, we detected substantial phosphorylated Erk (pErk) in wild-type SGCs, but not in SGCs of *fgf24* mutants (n = 13 and 11, respectively; Fig 4F–4G’). In contrast to *etv4* expression, pErk does not appear to be restricted to the inner layer of SGCs, indicating that *fgf24*-dependent Map kinase activity is present in apparently all SGC populations. These data therefore suggest that Fgf24 may activate *etv4* expression via the MAPK pathway, but that other factors must act to limit *etv4* expression to only the inner SGC.

### Fgf24 promotes differentiation and proliferation of the somatic gonad

**Fig 5:**
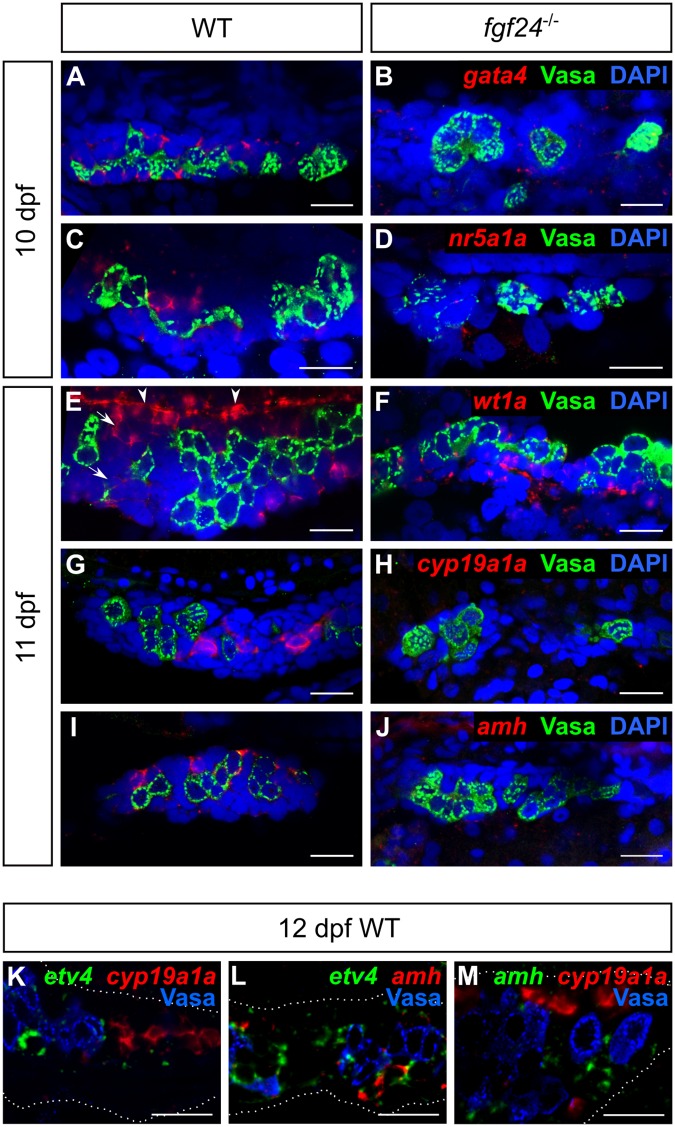
Fgf24 regulates gene expression of somatic gonad cells. (A-J) Single plane confocal micrographs of whole-mount larval gonads after fluorescent *in situ* hybridization detecting SGC markers (red). Nuclei labeled with DAPI (blue), germ cells labeled with Vasa (green). *gata4* (A), *nr5a1a* (C), *wt1a* (E), *cyp19a1a* (G), and *amh* (I) are expressed in somatic gonad cells (SGCs) of wild-type larval gonads, whereas only *wt1a* can be detected in SGCs of *fgf24* mutants (F). (K-M) Double fluorescent *in situ* hybridization and Vasa antibody staining (blue) of 12 dpf wild-type gonads (outlined by white dotted line). *etv4*, *cyp19a1a*, and *amh* are expressed in non-overlapping SGC domains (n = 12, 12, and 3 for K, L, and M, respectively). In E, arrows denote inner SGCs; arrowheads denote outer SGCs. A-M are sagittal optical sections with anterior to the left. Scale bars = 20 μm.

Our analysis thus far supports a model where the primary role of Fgf24 is to promote the development of the somatic gonad and that the loss of germ cells in *fgf24* mutants is a secondary consequence of defective somatic gonad development. To further this analysis, we analyzed the expression of two main classes of genes: 1) genes reportedly required for early gonad development in the mouse, and 2) genes known to be important for later gonad development and function in both mammals and fish. The transcription factors *Gata4*, Nuclear receptor subfamily 5 group a1 (*Nr5a1*, also called Steroidogenic factor 1, *Sf1*), and Wilms tumor protein 1 (*Wt1*) regulate early mouse gonad development. *Gata4* is required for epithelial proliferation during the initiation phase, while the latter two promote cell survival during the growth phase [11–14]. Using fluorescent RNA *in situ* hybridization we found that zebrafish *gata4* and *nr5a1a* orthologs are readily detectable in SGCs of wild-type 10 dpf gonads (n = 14 and 15, respectively), but absent or reduced in *fgf24* mutant gonads (n = 13 and 12, respectively; Fig 5A–5D). In contrast, we found that *wt1a*, one of two zebrafish *Wt1* orthologs, is expressed in the SGCs of both wild-type and *fgf24* mutant gonads at 11 dpf (n = 19 and 12, respectively; Fig 5E and 5F). Notably, *wt1a* appears to be expressed at lower levels in inner SGCs (arrows, Fig 5E) and most robustly in the outer layer of SGCs on the dorsal edge of the gonad (arrowheads, Fig 5E).

We next assessed the expression of genes associated with differentiated cell types of the larval gonad: *cyp19a1a*, which encodes an aromatase normally expressed in granulosa and theca cells of the adult ovary, and the Anti Müllarian Hormone-encoding gene *amh*, which is normally expressed in Sertoli cells of the adult testis [35, 36]. Because the larval gonad is initially bipotential, some genes that are later expressed sex-specifically, including *cyp19a1a* and *amh*, can be detected in the gonads of all animals during the early larval stages [35]. Indeed, we found that the expression of both genes can be detected in most wild-type gonads starting at 11 dpf (15/16, 16/16 respectively; Fig 5G and 5I). By contrast, the expression of these genes was not detected, or was detected at very low levels in gonads of 11 dpf *fgf24* mutants (2/12 and 1/11 showed low expression, respectively; Fig 5H and 5J). Because we found that two populations of somatic cells can be distinguished in the early larval gonad based on *fgf24* and *etv4* expression (Fig 4D), we performed high resolution fluorescent RNA *in situ* hybridization to determine in what cell layer *cyp19a1a* and *amh* are expressed. Similar to *etv4*, we found that both genes are expressed in only interiorly-localized cells. However, we did not detect co-expression of *cyp19a1a*, *amh*, or *etv4* in the same cells, indicating that the inner SGCs of 12 dpf gonads are comprised of at least three distinct cell populations (Fig 5K–5M). Together, these results further support the model that the primary function of Fgf24 is to promote development of SGCs.

**Fig 6:**
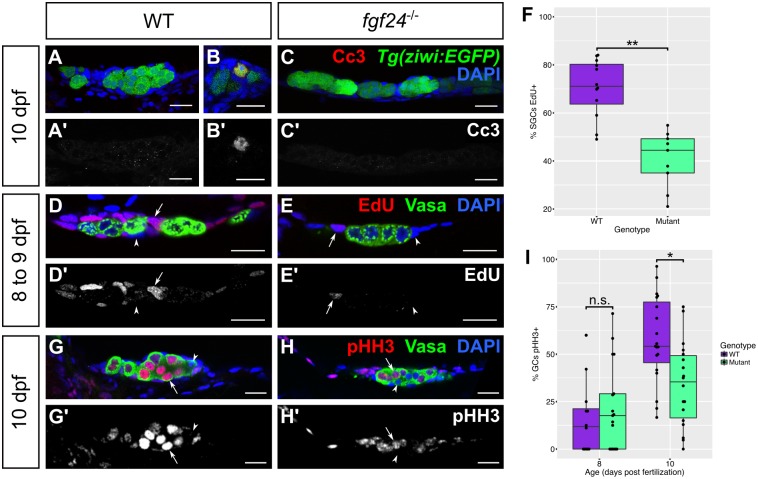
*fgf24* mutant somatic gonad cells and germ cells have low proliferation. (A-C’) 10 dpf wild-type (WT; n = 7; A, A’) and *fgf24* mutant (n = 7; C, C’) gonads exhibit rare Cc3 staining (red). Wild-type and *fgf24* mutant animals have similar numbers of Cc3(+) germ cells (mean ± SEM = 0.14 ± 0.14, 0, respectively; P = 0.36) and Cc3(+) SGCs (mean ± SEM = 0.57 ± 0.3, 0.43 ± 0.2, respectively; P = 0.7). (B, B’) In a 10 dpf wild-type gonad, one *Tg(ziwi*:*EGFP)* positive germ cell (green) stains positive for Cc3 (red). (D-F) EdU incorporation from 8 to 9 dpf. Fish exposed to 200 μM EdU between 8 and 9 dpf were euthanized and stained for Vasa (green) and EdU (red). A lower percentage of mutant somatic gonad cells have incorporated EdU compared to wild-type (mean = 41% and 70%, respectively; P < .001; arrow = EdU(+) SGC, arrowhead = EdU(-) SGC). (G-I) Antibody staining against phospho-Histone H3 (pHH3, red) and Vasa (green). At 8 dpf, germ cells of *fgf24* mutants are equally likely to be pHH3(+) as those of wild-type animals (mean = 22% and 16%, respectively; P > 0.05), however by 10 dpf, mutants have a significantly lower proportion of pHH3(+) germ cells compared to wild-type (mean = 34% and 58%, respectively; P < .01; arrow = EdU(+) germ cell, arrowhead = EdU(-) germ cell). Nuclei are labeled with DAPI (blue). A-E’, G-H’ are sagittal optical sections with anterior to the left. (F, I) Box and whisker plots depicting the percentage of proliferative cells in a gonad (purple = wild-type, green = *fgf24* mutant). Each dot represents the percentage of EdU(+) SGCs (F) or pHH3(+) GCs (I) in one gonad. (Unpaired two-tailed t-test; * = P < .01; ** = P < .001; n.s. = no significance). Scale bars = 20 μm.

In addition to the defects in somatic gonad gene expression, mutants older than 12 dpf have gonads that are smaller than their wild-type siblings because they have fewer germ cells and apparently fewer SGCs (Fig 2D–2F). Decreased cell numbers could be due to decreased cell proliferation, increased cell apoptosis, or a combination of the two. We first asked if mutant gonads have an increase in cell apoptosis relative to wild-type. We assessed the extent of apoptosis by staining for Cleaved caspase 3 (Cc3) and by performing a TUNEL assay. At 10 and 14 dpf, neither wild-type nor *fgf24* mutant gonads displayed appreciable apoptosis in either SGCs or germ cells (Fig 6A–6C’; S3A–S3B’ Fig). In addition, we asked if we could rescue the *fgf24* phenotype by blocking Tp53-mediated apoptosis. Using the *tp53* ^*M214K*^ allele [45] we produced *tp53;fgf24* double mutants, which phenocopied *fgf24* single mutants: 100% of double mutants were phenotypically male as adults and 69.2% lacked gonads completely (n = 13). In comparison, only 28.6% of *tp53* single mutants were male and 100% had two fully developed gonads (n = 14, S2 Table). Together, these data suggest that the decreased number of cells in *fgf24* mutant gonads is not a result of increased apoptosis.

Finally, we asked if decreased cell numbers in *fgf24* mutants was the result of reduced proliferation rates. We therefore exposed wild-type and *fgf24* mutant animals to the thymidine analog 5-ethynyl-2’-deoxyuridine (EdU) from 8 to 9 dpf to label cells in S-phase of the cell cycle. Although we detected EdU in SGCs of both genotypes, the percentage of SGCs that were EdU-positive was significantly higher in wild-type compared to mutant gonads (70% and 41%, respectively; P < .001; Fig 6D–6F). In contrast to SGCs, we never observed EdU incorporation in germ cells during this or later time frames (Fig 6D–6E’ and S4A–S4D’ Fig). Because there is a vast increase in germ cells during larval development (Fig 2D), we concluded that germ cells may incorporate this thymidine analog less efficiently, and therefore utilized an antibody against the mitosis-specific phospho-Histone H3 (pHH3) to identify germ cells in prophase, when pHH3 is detected throughout the nucleus [46]. While the percentages of pHH3-positive germ cells are similar between genotypes at 8 dpf, by 10 dpf wild-type gonads have a significantly higher proportion of pHH3-positive germ cells than mutant gonads (58% and 34%, respectively; P < .01; Fig 6G–6I). These results argue that decreased SGC and germ cell numbers in *fgf24* mutant gonads are due primarily to a decrease in cell proliferation.

### *fgf24* is required for morphogenesis of the larval gonad

**Fig 7:**
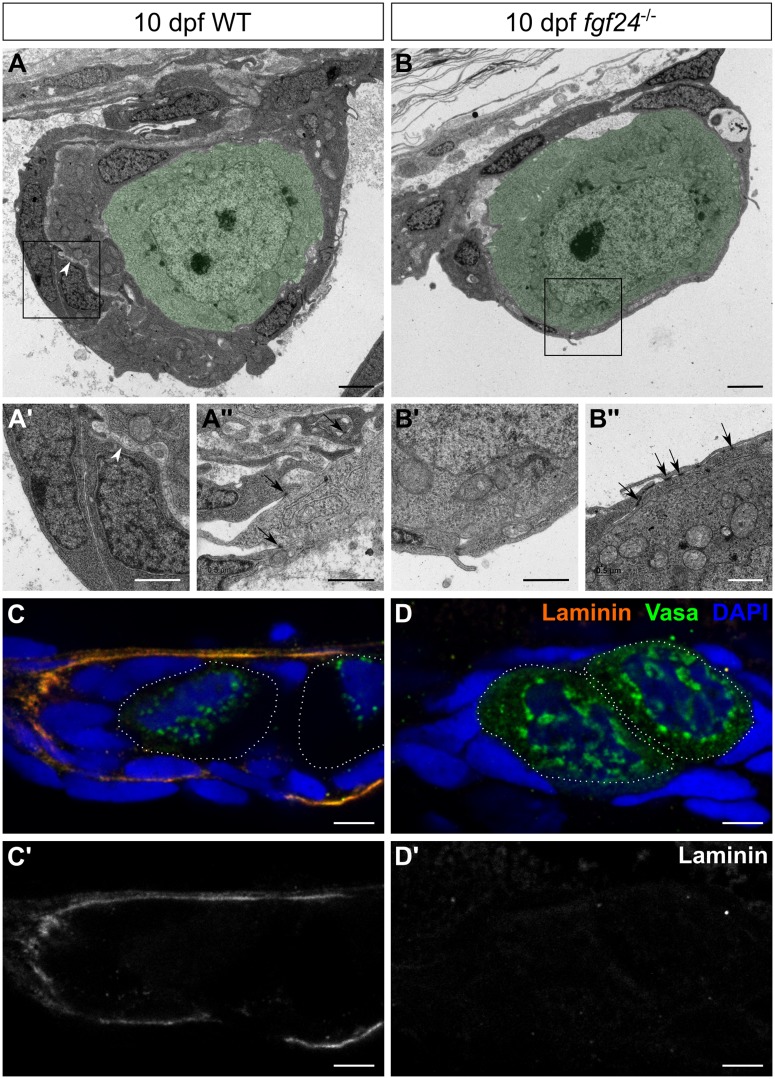
*fgf24* mutant gonads lack wild-type organization. (A-B”) Transmission electron micrographs of 10 dpf gonads, transverse sections. In both wild-type (WT) and *fgf24* mutant gonads, germ cells (pseudocolored green) are enclosed by SGCs (A, B). However, the somatic gonad component of the wild-type gonad is thicker than in the *fgf24* mutant gonad, and is split into two layers by an electron-lucent space (arrowheads in A and A’, which is a higher magnification of the box in A). The somatic gonad component is single layered, as shown at higher magnification in B’ (corresponds to the box in B). Somatic gonad cells extend processes and make cell-cell contacts (arrows) in both wild-type and *fgf24* mutant gonads (A”, B”, respectively). (C-D’) Antibody staining against Laminin (orange) and Vasa (green) of 10 dpf gonads; individual germ cells are outlined with dotted white lines. Laminin is deposited between nuclei of SGCs in wild-type (C, C’) but not *fgf24* mutant (D, D’) gonads. C-D’ are sagittal optical sections with anterior to the left. Nuclei are labeled with DAPI (blue). Scale bars = 2 μm in A, B; 1 μm in A’, A”, B’, B”; 5 μm in C-D’.

The data above indicate that the early zebrafish somatic gonad is composed of two somatic layers. It is therefore possible that the outer *fgf24*-expressing layer is a developing epithelium. To test this hypothesis, as well as to compare the overall structure of wild-type and *fgf24* mutant gonads at high resolution, we analyzed transverse sections by transmission electron microscopy (TEM). At 10 dpf, we found that both wild-type and mutant gonads were arranged with germ cells in the center, surrounded by SGCs (Fig 7A and 7B). However, the thickness of the SGC portion surrounding germ cells appeared to be greater in wild-type compared to mutant gonads. Furthermore, the SGCs in wild-type gonads were divided into two layers, likely corresponding to the *fgf24*-expressing cells and the *etv4*-expressing cells (Fig 7A). We noted that these layers were separated by an electron-lucent space, perhaps due to the presence of a basement membrane (n = 6; Fig 7A’). We therefore asked whether Laminin, a central component of the basal lamina, could be detected in this region. While Laminin is not detected in wild-type 8 dpf gonads (n = 8; S5A and S5A’ Fig), there is abundant Laminin deposited between the two layers of SGCs of wild-type 10 dpf gonads, indicating the presence of a basement membrane (n = 14; Fig 7C and 7C’). In contrast to wild-type, *fgf24* mutant gonads have only one layer of SGCs (n = 5; Fig 7B’) and lack Laminin staining altogether (n = 10; Fig 7D and 7D’). These results suggest that Fgf24 is required for normal morphogenesis of the early larval gonad.

Frequently, Fgf ligands are secreted by one cell layer (*e*.*g*. epithelial) and signal across a basement membrane to Fgf receptor-expressing cells of a second layer (*e*.*g*. mesenchymal; [47], reviewed in [48]). Analysis of our TEM data revealed that the outer layer of SGCs in wild-type gonads makes many cell-cell contacts, seen as electron dense patches, characteristic of epithelial cells (Fig 7A”). We therefore asked whether the *fgf24*- and *etv4*-expressing cells were adopting epithelial and mesenchymal fates, respectively. To address this question, we determined the cell junction landscape of each cell layer by staining for components of adherens and tight junctions.

**Fig 8:**
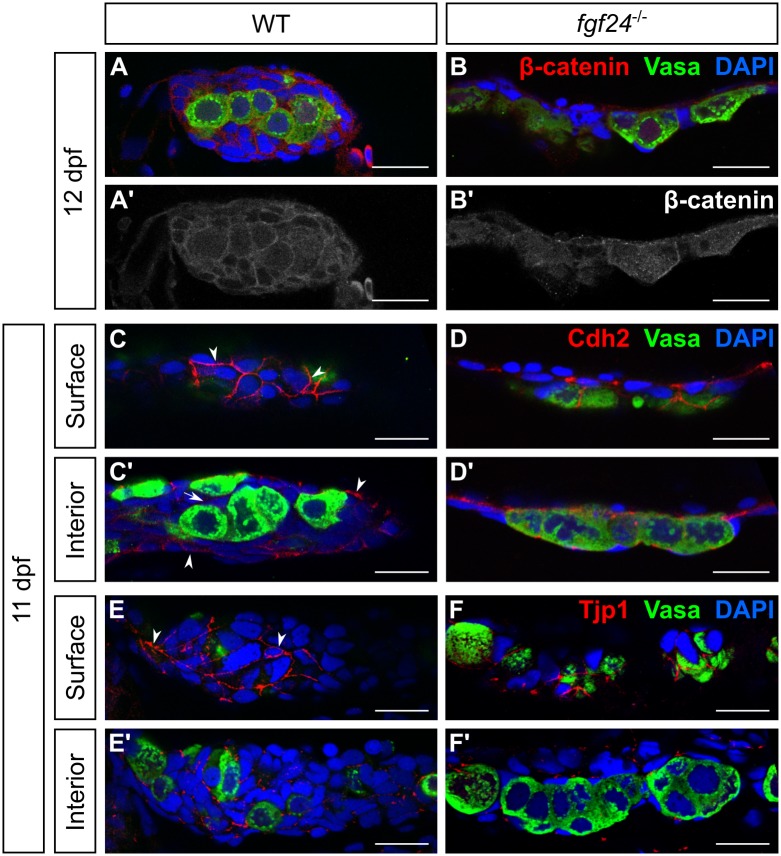
Somatic cells of wild-type and *fgf24* mutant gonads express epithelial-like cell adhesion molecules. (A-F’) Single plane confocal micrographs of whole-mount larval gonads immunostained for cell adhesion molecules (red). (A-B’) β-catenin localizes to the membranes of both SGCs and germ cells in wild-type (WT) gonads (A, A’). While β-catenin still localizes to the membranes of both cell types in *fgf24* mutants, it appears reduced (B, B’). (C-D’) Cdh2 localizes strongly to the outer layer of SGCs in wild-type and *fgf24* mutant gonads, visible on the surface of the gonad (C, D) and in the external-most layer of SGCs of the interior view (C’, D’). (E-F’) Similar to Cdh2, Tjp1 localizes to the outer layer of SGCs in wild-type (E, E’) and *fgf24* mutant (F, F’) gonads. A-F’ are sagittal optical sections with anterior to the left. Germ cells are labeled with Vasa (green), nuclei are labeled with DAPI (blue). Scale bars = 20 μm.

Adherens junctions are mediated by transmembrane cadherins, of which there are several types. Cadherin homodimerization helps similar cells associate with each other, and can promote cell sorting within a tissue. Inside the cell, catenins link the intracellular tail of the cadherin to actin, providing mechanical linkage between adjacent cells. In wild-type gonads, we found that β-catenin is expressed in virtually all cells, suggesting that all SGCs and germ cells have some type of adherens junctions (n = 7; Fig 8A and 8A’). Interestingly, we found that Cdh2/N-cadherin is highly localized to the membranes of the outer SGCs and weakly to the membranes of inner SGCs and germ cells (n = 17; arrowheads and arrows, respectively, Fig 8C and 8C’). In contrast, we see very low levels of Cdh1 (E-cadherin) in both germ cells and SGCs (n = 15; S6A–S6C’ Fig).

Tight junctions are a hallmark of epithelial cells, where they function to block the passage of fluids and molecules between cells. Tight junctions are formed by interactions between the transmembrane occludins and claudins and the intracellular Tjp1 (Tight junction protein 1/Zo-1), the latter of which interacts with actin. We found that Tjp1 was expressed in SGCs only and localized most intensely to the outer SGC membranes, similar to Cdh2 (n = 15; arrowheads, Fig 8E and 8E’). These data argue that the outer layer of *fgf24*-expressing SGCs forms an epithelium.

Our TEM data reveal that both wild-type and mutant SGCs make many electron-dense cell-cell contacts (arrows, Fig 7A” and 7B”). We therefore hypothesized that mutant gonads would also maintain the expression and localization of cell adhesion molecules. Indeed, we found that Cdh2 and Tjp1 remain strongly localized to some SGC membranes (n = 15 and 14, respectively; Fig 8D, 8D’ and 8F, 8F’), while β-catenin and Cdh1 appear slightly reduced (n = 7 and 10, respectively; Fig 8B and 8B’ and S6D and S6D’ Fig).

Finally, we sought to determine the identity of the SGCs that are present in the early *fgf24* mutant gonads. In wild-type animals, the outer, *fgf24*-expressing layer of SGCs showed strong localization of Cdh2 and Tjp1, the two cell adhesion molecules that were maintained in *fgf24* mutant somatic gonad cells. We therefore hypothesized that the SGCs that remain in the mutants are most similar to the *fgf24*-expressing epithelial cells of the wild-type gonad. To test this, we asked whether we could detect *fgf24* transcript in *fgf24* mutants, as it is known that nonsense mediated decay of transcripts with premature stop codons varies in efficiency [49, 50]. Using fluorescent *in situ* hybridization, we found that gonads of 11 dpf *fgf24* mutants have both SGCs with and without detectable *fgf24* transcripts (arrowheads and asterisks in S7A–S7B’ Fig, respectively). Thus, it appears that the *fgf24* mutant gonad, like the wild-type gonad, contains two distinct populations of SGCs, but that they fail to form the bi-layered organization observed in wild-type gonads.

## Discussion

Prior to this study, nothing was known about the genetic regulation of early somatic gonad development in zebrafish. Here we have presented evidence that early somatic gonad development in zebrafish is regulated, in part, by Fgf signaling mediated by the Fgf24 ligand. Our results show that *fgf24* is expressed in an outer layer of epithelial SGCs, and that these cells surround and signal to a distinct population of internally-located mesenchymal SGCs that are in close contact with the germ cells. In the absence of Fgf24 signaling, the inner SGCs fail to express genes known to be important for gonad development and/or maintenance. Additionally, our results suggest that Fgf24 signaling is required for the formation of this bi-layered early gonad. We find that while these early defects are fully penetrant in larval fish, as adults approximately 22% of animals eventually develop partial testes. Although the phenotypes we describe here are novel for mutants that affect the Fgf signaling pathway in any vertebrate, we argue that the requirement for Fgf signaling during the development of the early bipotential gonad may be widely conserved.

### The primary role of *fgf24* is to promote early somatic gonad development

**Fig 9:**
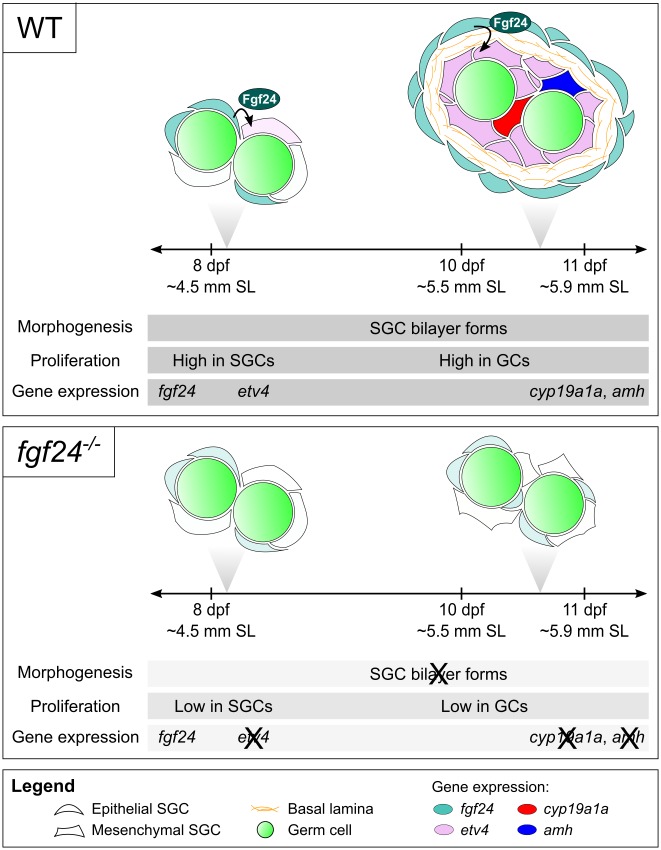
Cross-section model and timeline of wild-type and *fgf24* mutant gonad development. Gonads of wild-type (WT; top panel) and *fgf24* mutant animals (*fgf24*^-/-^; bottom panel) are initially similar (8 dpf). In wild-type gonads, *fgf24* (teal) is first expressed in a subset of SGCs at or slightly before 8 dpf. Shortly thereafter, *etv4* expression (lilac) is induced in a subset of *fgf24*(-) SGCs, and SGCs undergo proliferation. By 10 dpf, the somatic component of wild-type gonads is organized into a bilayer, whereby *fgf24*(+) reside in the outer epithelial layer and *etv4*(+) cells are situated in the inner SGC layer and in close contact with germ cells. At this point, most germ cells are now proliferative. By 11 dpf, subsets of the inner SGCs activate expression of either *cyp19a1a* (red) or *amh* (blue). In *fgf24* mutants, some *fgf24* message can be detected in a subset of SGCs (light teal). However, the inability to produce functional Fgf24 protein results in lower proliferation of SGCs and germ cells. Additionally, the somatic component fails to organize into a bilayer or initiate expression of *etv4*, *cyp19a1a*, or *amh*. Representative developmental staging of animals used in these experiments is reported as age (dpf) and estimated standard length (SL). SL was measured post-fixation (4% PFA), and estimated SL was calculated according to Parichy and colleagues ([53]; 8 dpf = 4.5 mm ± 0.023 (mean ± SEM; n = 20); 10 dpf = 5.5 mm ± 0.044 (n = 18); 11 dpf = 5.9 mm ± 0.052 (n = 18)).

We initiated these studies because of the discovery that all *fgf24* mutants are male as adults. This phenotype could suggest that the primary role of Fgf24 is to promote female sex determination or differentiation. However, our results strongly argue that the primary role of Fgf24 is instead to promote the development of the early bipotential gonad, the precursor to both ovaries and testes, and that the effect on sex determination is a secondary consequence of this primary defect. It is well established that germ cells, and in particular oocytes, are required for female sex determination and/or differentiation, as mutations that reduce or eliminate early germ cell development, or specifically early-stage oocytes during the bipotential phase, result in an all-male phenotype [32–34, 51, 52]. We have established here that all *fgf24* mutants have significantly reduced germ cells numbers relative to wild-type as early as 12 dpf (early bipotential stage), which can thus explain why all *fgf24* mutants develop as males. Importantly, we have presented evidence that gene expression and cell proliferation defects in the somatic gonad can be detected in *fgf24* mutants as early as 8 dpf, several days prior to when we can detect significant differences in germ cell numbers between wild-type and mutant larvae. Finally, the expression of both male- and female-specific genes is equally affected by loss of *fgf24* function (e.g. *amh* and *cyp19a1a*; Fig 9), a result that is inconsistent with Fgf24 having a sex-specific role. Thus, we strongly favor a model where the primary role of Fgf24 is in promoting somatic gonad development during the bipotential phase, and that defects in female development are a secondary consequence to earlier defects in the development of the bipotential gonad.

### How does Fgf signaling regulate gonad formation?

Our current data suggest that the gonads of *fgf24* mutants have fewer SGCs in comparison to their wild-type siblings. This phenotype could result from a failure in the specification and/or migration of somatic gonad precursors cells, a failure of these cells to proliferate, or a combination of these factors. In mammals, SGCs are derived from cells of the coelomic epithelium and, in males, there is also contribution from the mesonephric mesenchyme [54–56]. However, in fish, the origin of SGCs has so far only been investigated in Medaka. Using cell lineage-labeling, SGCs in Medaka have been shown to originate from the lateral mesoderm, which also likely includes precursors of the coelomic epithelium [57]. While similar studies have not been completed in zebrafish, a clear association of SGCs with germ cells can be detected in 5 dpf larvae, at a time when the primitive gonad is in direct contact with the adjacent coelomic epithelium lining the swim bladder [29]. This suggests that the coelomic epithelium is the likely source of SGCs in zebrafish. Although at present we cannot rule out the possibility that *fgf24* is involved in early specification of somatic gonad precursor cells, our analysis shows that SGCs are present in *fgf24* mutants, and that mutant cells, based on EdU incorporation, have significantly lower rates of proliferation than do wild-type SGCs. These data strongly argue that *fgf24* is necessary for the expansion of the SGC population, but not for their initial specification.

An interesting finding from this study is that even though the early larval somatic gonad in zebrafish is composed of relatively few cells, by 8 dpf at least two distinct cell populations can be identified. In wild-type animals, *fgf24* is expressed in most gonads by 8 dpf, but at this time point its expression does not appear to be spatially restricted. By 10 dpf, however, all *fgf24*-expressing cells are localized to an outer layer of SGCs surrounding an inner population of *fgf24*(-) cells. Coincident with this observation, formation of a basal lamina between these two layers is evident based on TEM and Laminin localization, and cells of the outer layer begin to express cell-junction components that are characteristic of epithelial cells (*e*.*g*. Cdh2 and Tjp1). Thus, it appears that by 10 dpf the zebrafish somatic gonad has organized into an inner mesenchymal-like layer of cells that are in direct contact with the germ cells, surrounded by an outer *fgf24*-expressing epithelial layer. Our data also suggest that development of the inner mesenchymal cells is dependent on Fgf24-mediated cell interactions with the epithelial layer reminiscent of the role of Fgf signaling in other developmental contexts (*e*.*g*. limb bud development). It is possible that the single cell layer present in 10 dpf *fgf24* mutant gonads represents only one of these two cell populations, but this has been difficult to determine as the expression of nearly all SGC marker genes so far examined is either greatly reduced or absent in mutant gonads. An exception to this is Wilms tumor 1a (*wt1a*), which appears to be expressed in both outer and inner SGCs in wild-type and in mutant gonads. This indicates that the expression of *wt1a* is independent of *fgf24* and therefore places Wt1a either upstream of, or in parallel with, Fgf24. In addition to *wt1a*, *fgf24* expression can be detected in a subset of the mutant SGCs suggesting that at least some of the remaining cells have an outer SGC-like characteristic, and consistently, analysis of cell-junction components suggests that a population of epithelial-like SGCs is still present in mutants. Thus, it appears that *fgf24* mutant gonads may contain at least two populations of somatic gonad precursor cells, but that these cells fail to mature into functional cell types in the absence of Fgf24 signaling.

In wild-type gonads, *etv4* appears to be expressed in most if not all of the inner somatic cells present in a wild-type gonad at 10 dpf. As development proceeds, the number of cells expressing *etv4* appears to decline, while at the same time, cells expressing differentiation marks such as *cyp19a1a* and *amh* increase in number. Interestingly, in 12 dpf gonads, we find little to no overlap between cells that express *etv4* and those that also express *cyp19a1a* or *amh*. Furthermore, *cyp19a1a*-expressing cells are also distinct from *amh*-expressing cells. These results together indicate that by 12 dpf, the inner population of SGCs is composed of at least three distinct cell populations: *etv4*(+), *cyp19a1a*(+), and *amh*(+). Given the dynamics of *etv4* expression relative to *cyp19a1a* and *amh*, we speculate that the *etv4*-expressing cells are a somatic gonad progenitor cell population that in turn gives rise to the differentiated functional cells of the gonad. If this is the case, the role of Fgf24 may therefore be to promote the development and proliferation of this progenitor population. Cell lineage analysis will be necessary to test this hypothesis.

### Conservation of Fgf signaling in vertebrate somatic gonad development

Thus far, no Fgf ligand has been shown to be necessary for mammalian bipotential gonad development analogous to the role we have described here for Fgf24 in zebrafish. Although it is possible that early gonad development in zebrafish is fundamentally different from that of tetrapods, there is reason to believe that this is not the case. Even though our understanding of gonad development in any teleost lags behind what is known in mammals, there are likely to be more similarities than differences in the genetic mechanisms that regulate gonad development and function in these two vertebrate lineages; many genes that are known to play essential roles during gonad development and sex determination in mammals are expressed at comparable time points during the development of the teleost gonad, and in some instances mutational analysis has confirmed their conserved roles. Examples include, but are not limited to: *Wt1*/*wt1a* ([58]; this report), *Nr5a1/nr5a1a* (*Steroidogenic factor 1*/*Sf1*; [59]; this report), *gata4* (this report), *Sox9*/*sox9a* [35, 60], and *dmrt1* [61, 62]. In mice, *Gata4*, *Nr5a1*, and *Wt1* are all required for early gonad development [11–14]. Molecular epistasis analysis has shown that *Gata4* is required for the expression of *Nr5a1*, but not *Wt1* [11]. Interestingly, we have shown that while all three genes are expressed in SGCs in wild-type zebrafish, *gata4* and *nr5a1a* transcripts are not detected in *fgf24* mutants, while *wt1a* expression appears to be normal. Thus, as in mice, the regulation of *wt1a* expression appears to be independent of *gata4* and *nr5a1a*.

An apparent exception to this conservation appears to be the role that Fgf signaling plays in gonad development, although in mammals, FGF9 and FGFR2 are known to play an important role during sex determination and differentiation [63–65]. Specifically, in mice, *Fgf9* is initially expressed in the sexually indifferent gonads of both sexes (starting as early as E9.5) after which its expression is stabilized only in the male gonad in response to expression of the male sex determinant *Sry* [64]. FGF9 both antagonizes the expression of WNT4, a female-promoting signal, and promotes the stable expression of the male-promoting SOX9 transcription factor ([66, 67]; reviewed in [68]). Disrupting the FGF9 signaling pathway leads to partial male-to-female sex reversal ([63, 64]; reviewed in [68]). However, similar to the *fgf24* mutant phenotype we present here, XY mice mutant for *Fgf9* experience a defect in SGC proliferation prior to the expression of *Sry*, signifying an earlier role for FGF9 in gonad development [62].

In addition to mice, the role of *FGF9* has also been investigated in chick. In both sexes of chick, *FGF9* is expressed first in the mesonephroi immediately adjacent to the early bipotential gonads and later in the epithelium that surrounds these bipotential gonads [69], a pattern that is strikingly similar to what we have reported here for *fgf24*. Furthermore, and regardless of sex, ectopic expression of FGF9 in the early chick gonad is sufficient to expand the apparent number of SGCs, while inhibition of FGF signaling using the Fgf receptor inhibitor SU5402 leads to an apparent reduction of SGCs. Collectively, these data argue that in tetrapods, FGF9 may function first during the formation of the early bipotential gonad in both sexes (similar to the role of Fgf24 reported here) and then again later to promote testis differentiation in males.

Finally, although there are striking similarities between the expression patterns and functions of *FGF9/Fgf9* in chick and mouse early gonads and that of *fgf24* in the early zebrafish gonad, Fgf24 and FGF9 belong to different FGF superfamilies: FGF9 is a member of the FGF9 superfamily, which includes FGF16 and FGF20, whereas Fgf24 is a member of the FGF8 superfamily, which includes FGF17 and FGF18 ([20, 23]; reviewed in [70]). It should be noted however that the FGF8 and FGF9 subfamilies are thought to have similar, though not identical, receptor binding specificity as measured in cell culture assays (reviewed in [70]). Interestingly, while genes encoding FGF9 and Fgf24 are both present in the genomes of representative basal ray-finned and lobed-finned fish (i.e. Spotted Gar and Coelacanth, respectively; [27, 71, 72]), FGF9 orthologs have not been found in any teleost genome to date, including zebrafish [73], and Fgf24 appears to have been lost in the lobed-fin lineage prior to the evolution of land dwelling tetrapods [23, 24]. An attractive hypothesis is that in animals with both genes, Fgf9 and Fgf24 function redundantly during early gonad development. If so, this could provide a means by which they could be lost after the divergence of the two main vertebrate lineages.

### Fgf signaling and ovarian cancer

Although a limited role for Fgf signaling in early mammalian gonadogenesis has been established, it is noteworthy that upregulation of the FGF-FGFR signaling pathway has been implicated as a causal factor for promoting certain types of aggressive ovarian cancers. For example, increased expression of each of the four mammalian Fgf receptors have been found in various epithelial ovarian cancers (EOC), and drugs that block or attenuate Fgf signaling have been shown to sensitize some EOCs to certain chemotherapeutic drugs [74–79]. In addition, the Fgf-responsive gene *Etv4* and its close family member *Etv5* are overexpressed in certain ovarian cancers [80–82]. It is therefore possible that, like in many cancers, ovarian cancers are caused, in part, by the unregulated activity of genetic pathways that are required for normal ovarian development during embryogenesis.

### *fgf24* is not required for adult male fertility

We have so far focused our attention on the role of Fgf24 during the development of the larval gonad. While all *fgf24* mutants have severe defects in larval gonad development, as adults 22% of *fgf24* mutants have partial testes that can produce functional sperm. Although it is not known how the mutant gonads resume apparently normal development, it is clear that this development can occur in the absence of Fgf24 function. One explanation for this phenomenon is that Fgf signaling is not involved in gonad formation at later stages; however it is also plausible that a second Fgf ligand expressed during juvenile development can rescue somatic gonad development in the absence of Fgf24. If this is the case, then latent testis development could occur in mutants that retain germ cells until expression of this ligand initiates. Future experiments will be necessary to test these models.

### Conclusion

Despite the important role the vertebrate somatic gonad plays in protecting germ cells and in regulating their maintenance and differentiation into gametes, little is known about the genetic regulation of somatic gonad development, and, in particular, the cell-cell interactions that are necessary for its development. Here, we have identified the Fibroblast growth factor ligand Fgf24 as a key player in this process. These results help to establish the zebrafish as a model for understanding the genetic regulation of early somatic gonad development in vertebrates.

## Materials and methods

### Ethics statement

The University of California Davis IACUC approved all animals used in this study (protocol #18483), and all animals used were euthanized using the American Veterinary Medical Association-approved method of hypothermal shock.

### Husbandry and fish strains

The wild-type strain *AB was used for the generation of *fgf24*^*uc47*^. Zebrafish husbandry was performed as previously described [83], with the following modifications to the larval fish (5-30dpf) feeding schedule: 5-12dpf: 40 fish/250mL in static fish water (4parts/thousand (ppt) ocean salts) were fed rotifers (*Brachionus plicatilis*, L-type) twice daily *ad libitum*. 12–15 dpf: 40 fish/ 1 liter gently flowing fish water (<1ppt ocean salts) were fed both rotifers and freshly hatched *Artemia* nauplii *ad libitum* twice daily. 15-30dpf: 40 fish/1 liter gently flowing fish water (<1ppt ocean salts) were fed freshly hatched *Artemia* nauplii *ad libitum* twice daily. The following alleles were used in this study: *fgf24*^*hx118*^, *fgf24*^*uc47*^, *tp53*^*zdf1*^. The following transgenic lines were used: *Tg(ziwi*:*EGFP)*^*uc02*^, *Tg(gsdf*:*mCherry)*^*uc46*^.

### Generation of *fgf24*^*uc47*^

The sgRNA and *cas9* mRNA components were produced as previously described [84]. Briefly, the sgRNA was designed to target exon 3 of *fgf24* (zifit.partners.org/ZiFiT/). Two oligonucleotides (5’-TAGGCAAGAAGATTAACGCCAA-3’ and 5’-AAACTTGGCGTTAATCTTCTTG-3’) were annealed and cloned into plasmid pDR274 (Addgene Plasmid #42250). The plasmid was linearized with DraI, and *in vitro* transcription was performed with the T7 polymerase (Roche, Cat. No. 10881775001). The *cas9*-expressing pMLM3613 plasmid was also obtained from Addgene (Plasmid #42251) and mRNA synthesis was performed as described [84]. The sgRNA and *cas9* mRNA were coinjected into one-cell embryos with phenol red (5% in 2M KCl) at a concentration of 12.5 ng/μL and 300 ng/μL, respectively. CRISPR efficiency was determined by comparing the DNA isolated from eight injected embryos with eight uninjected control embryos (24 hpf) using High Resolution Melt Analysis (HRMA) as described [85] (the primers used are listed in S1 Methods). At three months post-injection, germline mutations were identified from extracted sperm of injected males by PCR analysis and gel electrophoresis. PCR products with evident indels were cloned into the pGEM-T Easy vector (Promega, Cat. No. A137A) and sequenced. The individual containing the *fgf24*^*uc47*^ allele was outcrossed to *AB to obtain a heterozygous line.

### Genotyping

Fish were genotyped by extracting gDNA from caudal fin tissue. *fgf24*^*uc47*^, *Tg(ziwi*:*EGFP)*^*uc02*^, and *Tg(gsdf*:*mCherry)*^*uc46*^ were assayed using standard PCR conditions and gel electrophoresis. *fgf24*^*hx118*^ and *tp53*^*zdf1*^ were analyzed using HRMA. See S1 Methods for primer sequences.

### Fertilization tests

Male fish were euthanized in an ice water bath. Testes were removed and macerated with scissors in Hank’s solution [83]. Eggs were squeezed from wild-type females according to standard protocols [81] and were fertilized with 30 μL sperm from either wild-type or *fgf24*^*-/-*^ males. After three hours, the numbers of fertilized and unfertilized eggs were recorded.

### RNA *in situ* hybridization

RNA probes that detect the following genes were used: *cyp19a1a* and *amh* [33]; *gata4* [86]; *wt1a* [58]; *etv4* [87]. For all other plasmids for probe synthesis, mRNA was isolated from 24 hpf embryos, adult testis, or ovary using TRI reagent (Sigma-Aldrich, Cat. No. T9424) and synthesized into cDNA using the RETROScript Reverse Transcription Kit (ThermoFisher, Cat. No. AM1710). Targets were PCR amplified with Takara Ex Taq (Clontech, Cat. No. RR001A) and primers found in S1 Methods, Table 2. PCR products were cloned into an appropriate vector, and plasmids were linearized using endonucleases from New England Biolabs (see S1 Methods for details). *in vitro* transcription yielded antisense probes (Roche T7 or SP6 RNA polymerases (Cat. Nos. 10881775001 or 10810274001, respectively)). Probes were ethanol precipitated and G-50 sephadex column purified to remove excess nucleotides (GE Healthcare, Cat. No. 45-001-398). Probes were used at a concentration of 0.5-2ng/μL in hybridization solution.

Fish were fixed in 4% paraformaldehyde (PFA) overnight at 4°C or 4 hours at room temperature. Samples were transferred to 100% methanol and stored at -20°C for at least 16 hours. Samples were bleached for 10–15 minutes, as needed, prior to proteinase K digestion in 3% H_2_O_2_, 0.5% KOH. Color *in situ* hybridizations were performed similar to Thisse and Thisse, 2008 [88], with the exception that 5% dextran sulfate was included in the hybridization solution. Fluorescent *in situ* hybridizations were performed as in Lauter et al., 2011 [89]. Tyramide reactions were performed with commercially available tyramides (Life Technologies, Cat. Nos. T20948, T20950, and T20951). Gonads were dissected, and DNA was stained with DAPI. Samples were dehydrated by an increasing glycerol gradient. Gonads were mounted whole and imaged with an Olympus FV1000 laser scanning confocal microscope.

### Immunofluorescence

Tissue was prepared and treated as for ISH. After initial washing, nonspecific antibody was blocked with 2% BSA and 2% goat serum in PBS-DT (1% PBS + 0.1% Triton-X + 1% DMSO) for one hour at room temperature. Antibodies were diluted in blocking solution according to S1 Methods and applied to tissue overnight at 4°C. Following washing, blocking was repeated. Alexa Fluor secondary antibodies (Thermo Fisher Scientific, Cat. nos. A-11008, A-11012, A-11005, A-11001, A-11039, A-11042) were diluted at 1:500 in blocking solution and incubated with tissue overnight at 4°C to detect primary antibodies. DNA was stained with DAPI, and samples were dehydrated by an increasing glycerol gradient. Gonads were dissected, mounted whole, and imaged with an Olympus FV1000 laser scanning confocal microscope.

For the experiment shown in Fig 6G–6H’, an anti-Mouse-HRP conjugated secondary antibody (ThermoFisher, Cat. no. G-21040) was used to detect anti-pHH3, and tyramide reactions were performed as described above. For the experiment shown in Fig 7C and 7D, both Laminin and Vasa antibodies were raised in rabbit. Staining was therefore performed as above, but sequentially; briefly, samples were incubated overnight at 4°C with Rabbit anti-Laminin, washed, and incubated with a Goat anti-Rabbit IgG, Alexa Fluor 488 overnight at 4°C. After extensive washing, samples were incubated overnight at 4°C with Rabbit anti-Vasa, washed, and incubated with a Goat anti-Rabbit IgG, Alexa Fluor 594 overnight at 4°C. Samples were then treated as above.

### TUNEL assay

Apoptosis was detected with the ApopTag Apoptosis Detection Kit (Millipore, Cat. No. S7110). Samples were treated according to the manufacturer’s manual, with additional post-fixation steps after proteinase K digestion: Samples were treated with 4% PFA for 20 minutes at room temperature, washed 5 X 5 minutes in PBSTw (PBS + 0.1% Tween-20), incubated in pre-chilled 2:1 EtOH:acetic acid for 10 minutes at -20°C, and washed 3 X 5 minutes in PBSTw.

### EdU incorporation

Larvae were allowed to swim freely in 200 μM EdU + 0.1% DMSO. To diminish any systemic affects of treatment, fish were kept at normal densities and on normal feeding schedules. Fish were euthanized and fixed in 4% PFA immediately following exposure. After extensive washing in PBSTw, EdU was detected by “click” chemistry (10 μM Alexa Fluor 594 Azide (ThermoFisher, Cat. no. A10270), 1 mM CuSO_4_, 100 mM Tris pH8.5, 100 mM Ascorbic acid; incubate for 30 minutes at room temperature) and visualized on an Olympus FV1000 laser scanning confocal microscope.

### Cell quantification

Sagittal optical sections were collected at 5 μm intervals throughout the entirety of whole-mount gonads with an Olympus FV1000 laser scanning confocal microscope. Intervals of 5 μm were used to allow for identification of virtually every cell in a gonad. Individual cells were manually documented with the Cell Counter plugin for FIJI. Germ cells were identified by Vasa expression. Because we lack a pan-SGC marker and because gonads of the stages described here do not readily dissect from the body wall, we were not confident in our ability to quantify the total number of SGCs in any given gonad. However, for the EdU experiment, we were able to roughly identify SGCs based on their proximity to germ cells and overall shape of the tissue. Once somatic cells of a gonad were identified and recorded, overlap of EdU signal was scored.

### Transmission electron microscopy

*Tg(ziwi*:*gfp)*^*uc02*^ fish were euthanized in an ice water bath. Tails were removed immediately posterior to the gonads as visualized by the germ cell-specific GFP. They were then fixed 24 hours in Karnovsky's fixative (2.5% glutaraldehyde + 2% paraformaldehyde in 0.1 M sodium cacodylate), following 2 X 15 min rinses in 0.1 M sodium cacodylate buffer. Samples were then treated with 2% osmium tetroxide for 1 hour, followed by 2 X 15 minutes rinses in 0.1 M sodium cacodylate buffer. Tissue was dehydrated in an ethanol gradient (30 minutes each: 50%, 75%, 95% EtOH; 2 X 20 minutes 100% EtOH) and treated with propylene oxide 2 X 10 minutes. Tissue was pre-infiltrated with 1:1 propylene oxide:Poly/Bed 812 resin overnight and infiltrated with 100% Poly/Bed 812 resin for three hours (Polysciences, Inc). Finally, samples were embedded in fresh resin, polymerized in a 60°C oven for 24 hours, and sectioned to 100 nm. Sections were imaged using a Philips BioTwin CM120 TEM.

### Image and statistical analysis

Images were analyzed using FIJI, and only linear manipulations of brightness and contrast were applied. Statistical analysis and graphing were completed in R using standard packages, ggplot2, and ggbeeswarm.

## Supporting information

S1 Fig A fraction of *fgf24* mutants undergo latent testis development. (A-B) Dissected testes from 3.5 mpf wild-type (WT; A) and *fgf24* mutant (B) animals, anterior to the left. (C, D) Confocal images of isolated *Tg(ziwi*:*EGFP)* (green) and DAPI (blue) stained 4 mpf wild-type (C) and mutant (D) testes. Sg = spermatogonia; Sc = spermatocytes; Sz = mature spermatozoa; Sgc = somatic gonad cells. (E-H) *in situ* hybridization showing expression of *amh* (E, F) and *gsdf* (G, H) in 6 mpf wild-type (E, G) and mutant (F, H) testes. (I) Schematic diagram of the full-length Fgf24 protein and the predicted truncated peptides resulting from the *fgf24*^*hx118*^ and *fgf24*^*uc47*^ alleles. SP = signal peptide; hatching indicates missense amino acids. Scale bars = 20 μm.
(TIF)

S2 Fig Gsdf is expressed in somatic cells of the 32 dpf ovary. (A) By 32 dpf, wild-type ovaries have many germ cells, both premeiotic and oogenic (Oo), that are surrounded by *Tg(gsdf*:*mCherry)* positive granulosa cells (red; nuclei labeled with DAPI, blue). The boxed nucleus is magnified in the inset, with an arrow indicating the large nucleolus (DAPI only, in grey). Scale bar = 20 μm.
(TIF)

S3 Fig Gonads of wild-type and *fgf24* mutant animals have low levels of TUNEL incorporation at 14 dpf. (A-B’) TUNEL incorporation and Vasa staining of 14 dpf gonads. Both wild-type (WT; n = 5; A, A’) and *fgf24* mutant (n = 5; B, B’) gonads show similarly low levels of TUNEL staining (red, arrowheads). A-B’ are sagittal optical sections with anterior to the left. Germ cells are labeled with Vasa (green), nuclei are labeled with DAPI (blue). Scale bars = 20 μm.
(TIF)

S4 Fig Larval germ cells do not incorporate EdU. (A-B’) Single plane confocal micrographs of whole-mount wild-type larval gonads showing EdU incorporation (red). Larvae were allowed to swim freely in 200 μM EdU + 0.1%DMSO from 10 to 11 dpf (A, A’) or 12 to 13 dpf (B, B’), euthanized, fixed, and processed for detection of EdU. Many SGCs are EdU-positive at both timepoints, while germ cells are consistently EdU-negative. Germ cells are labeled with Vasa (green) and nuclei are labeled with DAPI (blue). A’ and B’ show the EdU channel only, in grey. A,-B’ are sagittal optical sections with anterior to the left. Scale bars = 20 μm.
(TIF)

S5 Fig Basal laminae are absent from 8 dpf wild-type gonads. (A, A’) Single plane confocal micrographs of whole-mount larval gonads immunostained for Laminin (red) and Vasa (green). Laminin is undetectable in either merged (A) or Laminin-only channel (A’), suggesting that basal laminae have not formed. A, A’ are sagittal optical sections with anterior to the left. Nuclei are labeled with DAPI (blue). Scale bars = 20 μm.
(TIF)

S6 Fig Wild-type and mutant gonads have low levels of membrane-associated Cdh1/E-cadherin at 10 dpf. (A-D’) Single plane confocal micrographs of whole-mount larval gonads immunostained for Cdh1/E-Cadherin. In most 10 dpf wild-type (WT; A, A’; 10/15) and *fgf24* mutant (D, D’; 10/10) animals, Cdh1 (red) does not localize to cell membranes of gonadal cells. In some cases, wild-type animals have low expression of Cdh1 at the membranes of SGCs (B, B’; 3/15) or germ cells (C, C’; 2/15). A-D’ are sagittal optical sections with anterior to the left. Germ cells are labeled with Vasa (green), nuclei are labeled with DAPI (blue). (A’, B’, C’, D) Cdh1 channel only, in grey. Arrow = membrane localization of Cdh1 in gonadal cells; Asterisk = membrane localization of Cdh1 in a nearby, non-gonadal tissue. Scale bars = 20 μm.
(TIF)

S7 Fig *fgf24* expressing and non-expressing somatic cells are present in the gonads of *fgf24* mutants. (A-B’) Single plane confocal micrographs of whole-mount larval gonads after fluorescent *in situ* hybridization. *fgf24* mRNA (red) can be detected in some, but not all, SGCs of both wild-type (WT; A, A’) and *fgf24* mutant (B, B’) animals at 11 dpf. A-B’ are sagittal optical sections with anterior to the left. Germ cells are labeled with Vasa (green), nuclei are labeled with DAPI (blue). Arrowhead = *fgf24*-positive SGC; Asterisk = *fgf24*-negative SGC. Scale bars = 20 μm.
(TIF)

S1 Table *fgf24* males produce functional sperm. Sperm isolated from three wild-type (WT) and three *fgf24* mutant males were able to fertilize eggs from wild-type females with similar efficiencies. (Unpaired two-tailed t-test, P = 0.835).
(TIF)

S2 Table p53-mediated apoptosis is not responsible for the *fgf24* mutant phenotype. Results from two separate experiments. In both experiments, *tp53*^+/-^;*fgf24*^+/-^ fish were incrossed and grown until 2 or 4 mpf. Euthanized fish were fixed and phenotyped for sex, and gonads were dissected and counted. Fish were then genotyped for both *tp53* and *fgf24*. Since all genotypes were reared together, we would expect similar sex ratios. Unlike *tp53* single mutants, however, *tp53*;*fgf24* double mutants are all phenotypic males usually with zero or one gonad, similar to *fgf24* single mutants.
(TIF)

S1 Methods Genotyping primers, *in situ* probe synthesis details, antibody specifications. (DOCX)
