# Supplementary figures and images for: Fibroblast growth factor signaling is required for early somatic gonad development in zebrafish
Source: PLoS Genet. 2017 Sep 5;13(9):e1006993. doi: 10.1371/journal.pgen.1006993 (PMC5600409; doi:10.1371/journal.pgen.1006993)

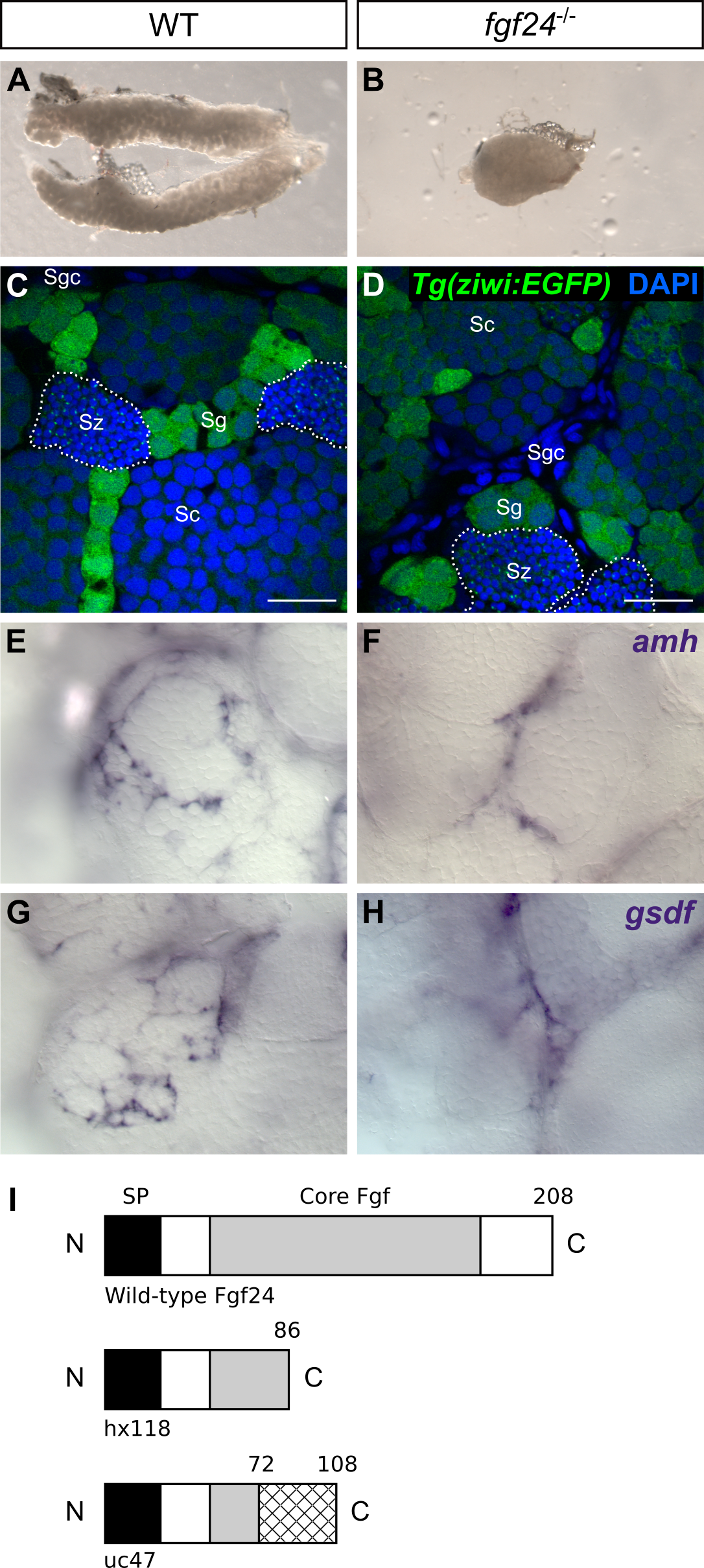

Supplement: S1 Fig — (A-B) Dissected testes from 3.5 mpf wild-type (WT; A) and fgf24 mutant (B) animals, anterior to the left. (C, D) Confocal images of isolated Tg(ziwi:EGFP) (green) and DAPI (blue) stained 4 mpf wild-type (C) and mutant (D) testes. Sg = spermatogonia; Sc = spermatocytes; Sz = mature spermatozoa; Sgc = somatic gonad cells. (E-H) in situ hybridization showing expression of amh (E, F) and gsdf (G, H) in 6 mpf wild-type (E, G) and mutant (F, H) testes. (I) Schematic diagram of the full-length Fgf24 protein and the predicted truncated peptides resulting from the fgf24hx118 and fgf24uc47 alleles. SP = signal peptide; hatching indicates missense amino acids. Scale bars = 20 μm. (TIF) [file pgen.1006993.s001.tif]

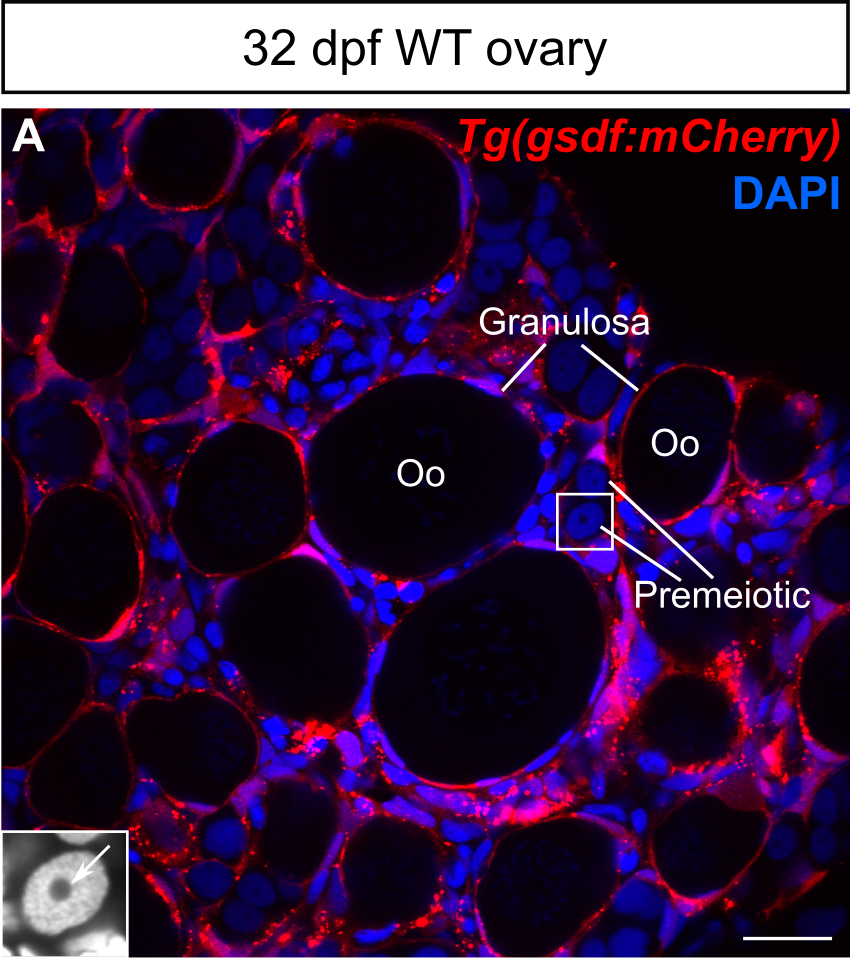

Supplement: S2 Fig — (A) By 32 dpf, wild-type ovaries have many germ cells, both premeiotic and oogenic (Oo), that are surrounded by Tg(gsdf:mCherry) positive granulosa cells (red; nuclei labeled with DAPI, blue). The boxed nucleus is magnified in the inset, with an arrow indicating the large nucleolus (DAPI only, in grey). Scale bar = 20 μm. (TIF) [file pgen.1006993.s002.tif]

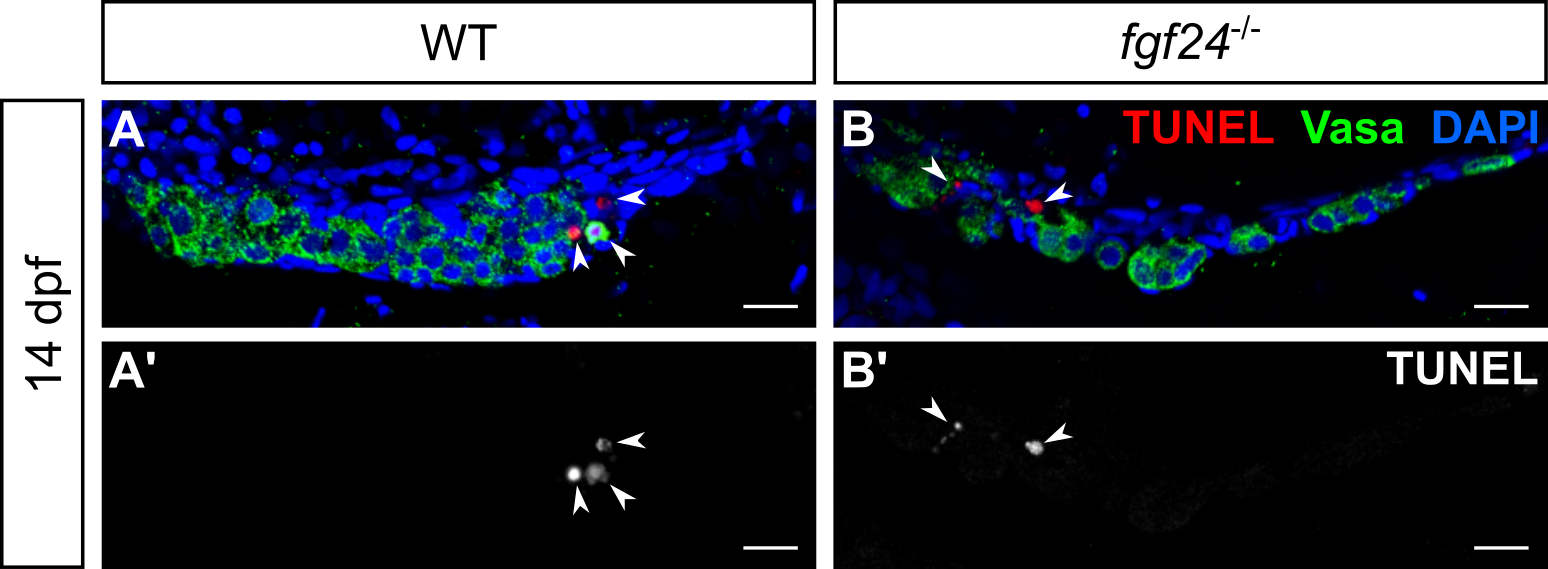

Supplement: S3 Fig — (A-B’) TUNEL incorporation and Vasa staining of 14 dpf gonads. Both wild-type (WT; n = 5; A, A’) and fgf24 mutant (n = 5; B, B’) gonads show similarly low levels of TUNEL staining (red, arrowheads). A-B’ are sagittal optical sections with anterior to the left. Germ cells are labeled with Vasa (green), nuclei are labeled with DAPI (blue). Scale bars = 20 μm. (TIF) [file pgen.1006993.s003.tif]

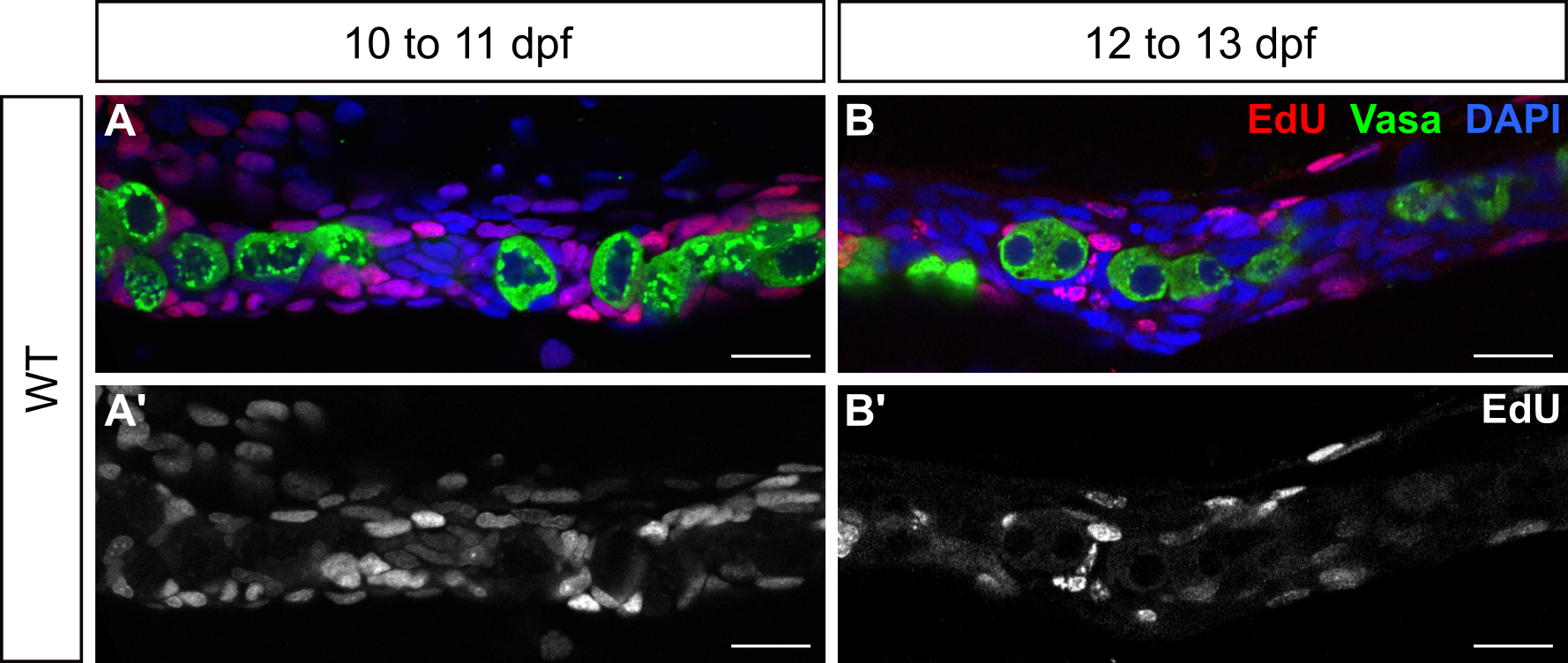

Supplement: S4 Fig — (A-B’) Single plane confocal micrographs of whole-mount wild-type larval gonads showing EdU incorporation (red). Larvae were allowed to swim freely in 200 μM EdU + 0.1%DMSO from 10 to 11 dpf (A, A’) or 12 to 13 dpf (B, B’), euthanized, fixed, and processed for detection of EdU. Many SGCs are EdU-positive at both timepoints, while germ cells are consistently EdU-negative. Germ cells are labeled with Vasa (green) and nuclei are labeled with DAPI (blue). A’ and B’ show the EdU channel only, in grey. A,-B’ are sagittal optical sections with anterior to the left. Scale bars = 20 μm. (TIF) [file pgen.1006993.s004.tif]

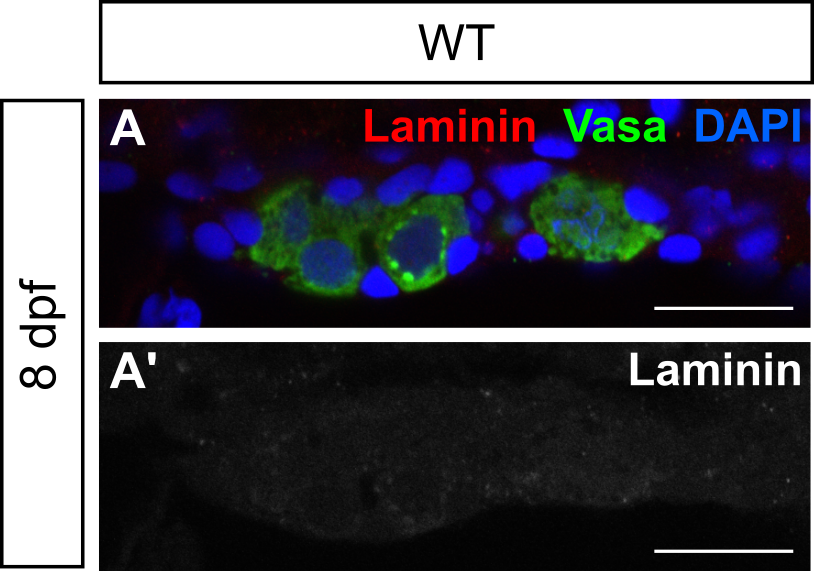

Supplement: S5 Fig — (A, A’) Single plane confocal micrographs of whole-mount larval gonads immunostained for Laminin (red) and Vasa (green). Laminin is undetectable in either merged (A) or Laminin-only channel (A’), suggesting that basal laminae have not formed. A, A’ are sagittal optical sections with anterior to the left. Nuclei are labeled with DAPI (blue). Scale bars = 20 μm. (TIF) [file pgen.1006993.s005.tif]

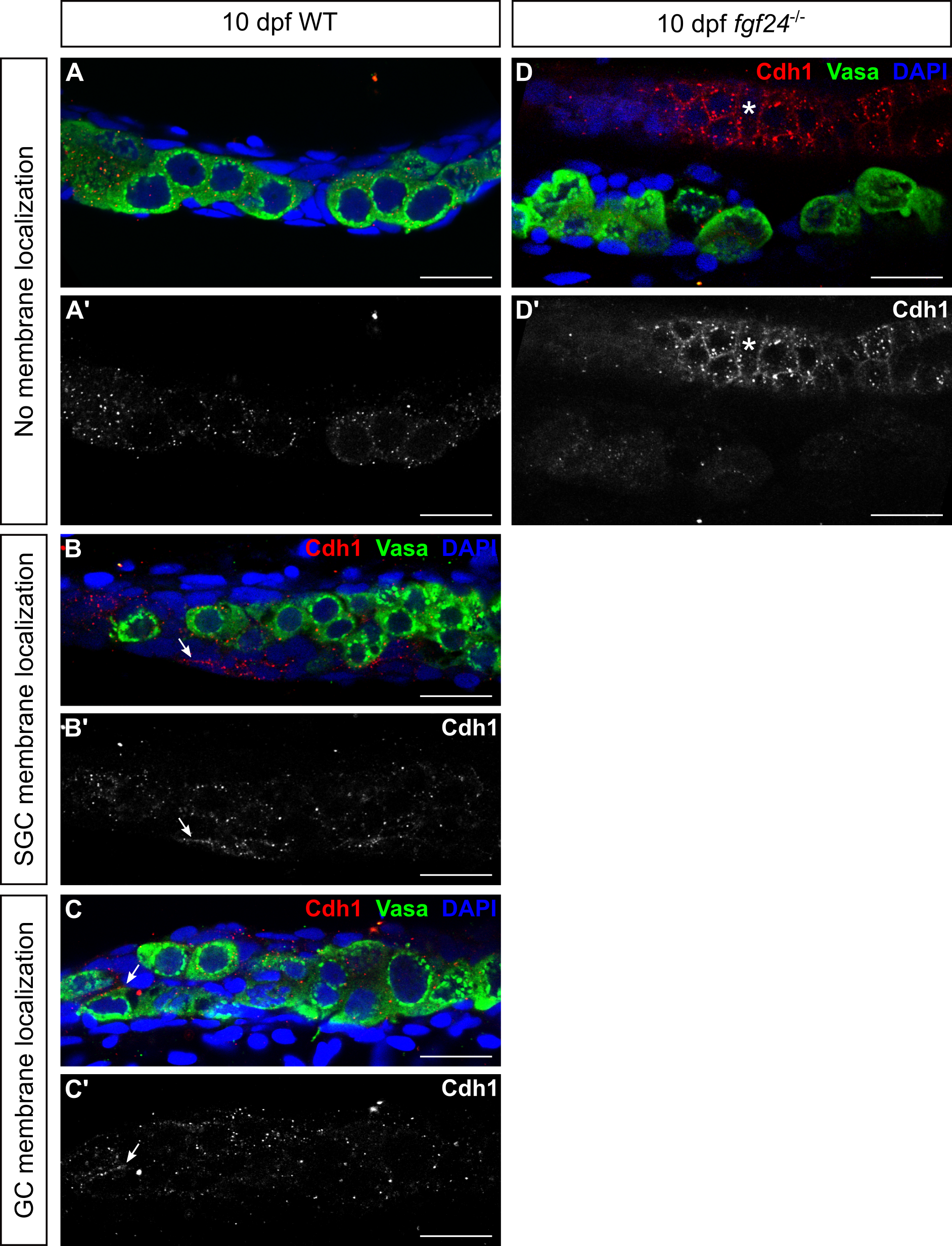

Supplement: S6 Fig — (A-D’) Single plane confocal micrographs of whole-mount larval gonads immunostained for Cdh1/E-Cadherin. In most 10 dpf wild-type (WT; A, A’; 10/15) and fgf24 mutant (D, D’; 10/10) animals, Cdh1 (red) does not localize to cell membranes of gonadal cells. In some cases, wild-type animals have low expression of Cdh1 at the membranes of SGCs (B, B’; 3/15) or germ cells (C, C’; 2/15). A-D’ are sagittal optical sections with anterior to the left. Germ cells are labeled with Vasa (green), nuclei are labeled with DAPI (blue). (A’, B’, C’, D) Cdh1 channel only, in grey. Arrow = membrane localization of Cdh1 in gonadal cells; Asterisk = membrane localization of Cdh1 in a nearby, non-gonadal tissue. Scale bars = 20 μm. (TIF) [file pgen.1006993.s006.tif]

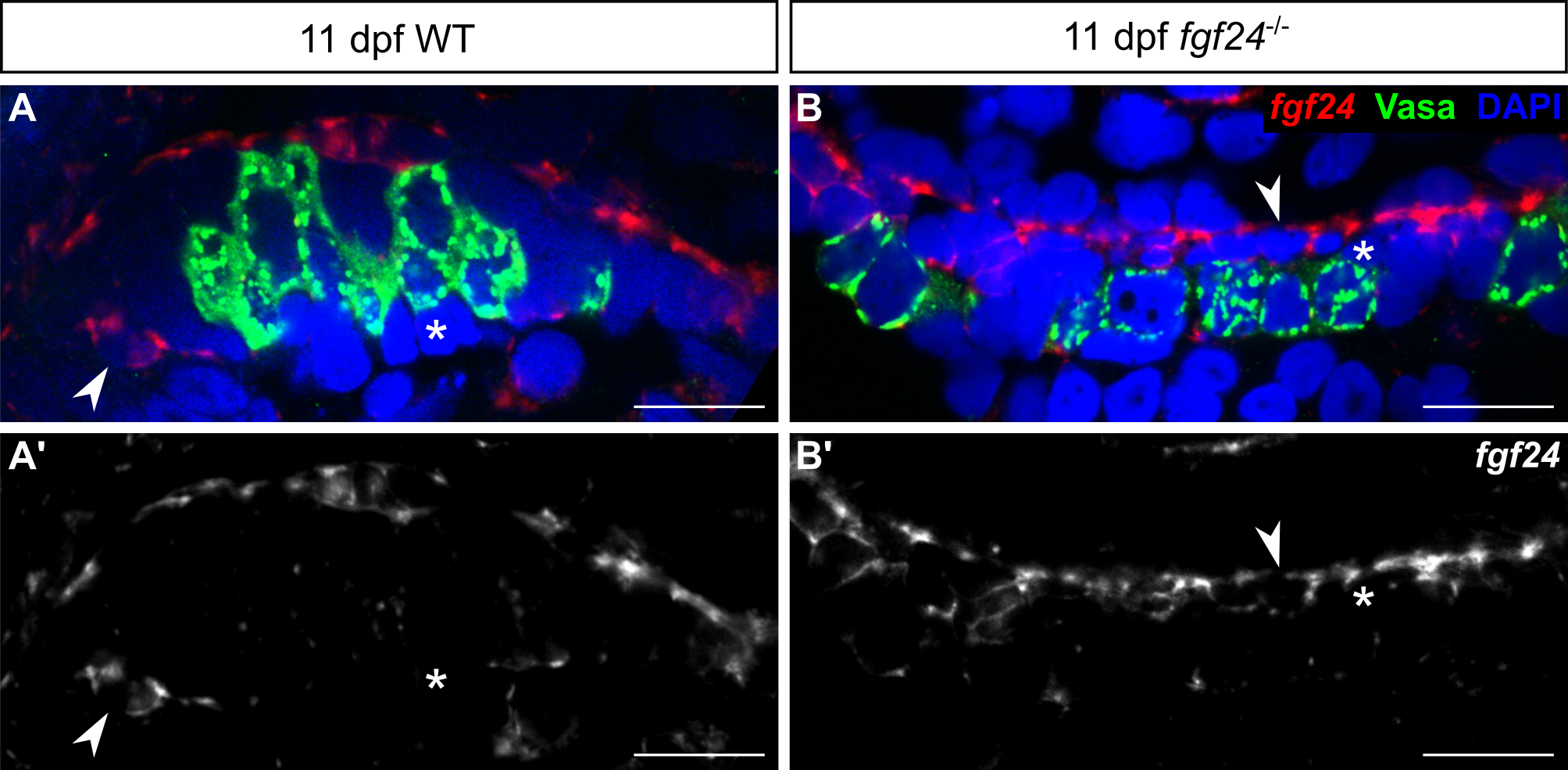

Supplement: S7 Fig — (A-B’) Single plane confocal micrographs of whole-mount larval gonads after fluorescent in situ hybridization. fgf24 mRNA (red) can be detected in some, but not all, SGCs of both wild-type (WT; A, A’) and fgf24 mutant (B, B’) animals at 11 dpf. A-B’ are sagittal optical sections with anterior to the left. Germ cells are labeled with Vasa (green), nuclei are labeled with DAPI (blue). Arrowhead = fgf24-positive SGC; Asterisk = fgf24-negative SGC. Scale bars = 20 μm. (TIF) [file pgen.1006993.s007.tif]

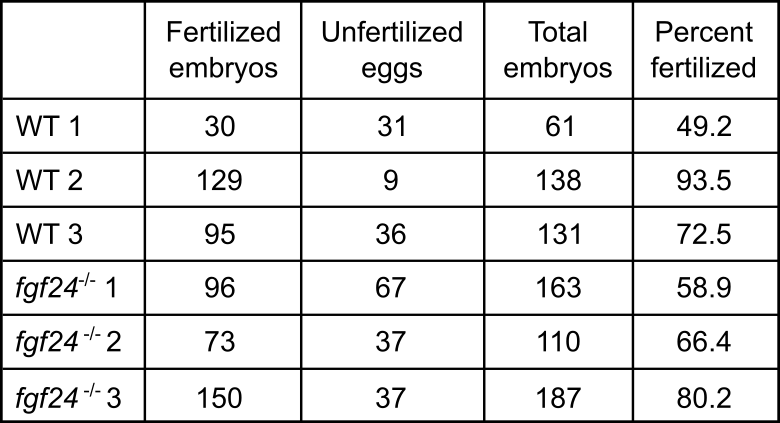

Supplement: S1 Table — Sperm isolated from three wild-type (WT) and three fgf24 mutant males were able to fertilize eggs from wild-type females with similar efficiencies. (Unpaired two-tailed t-test, P = 0.835). (TIF) [file pgen.1006993.s008.tif]

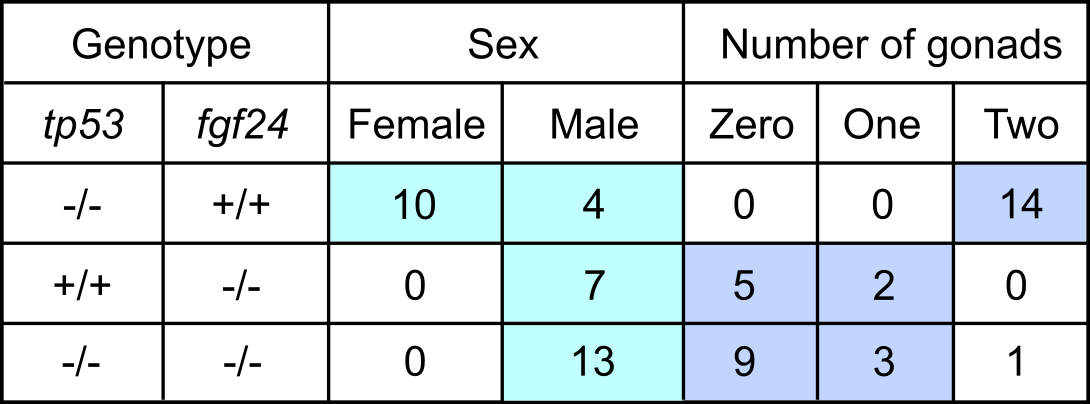

Supplement: S2 Table — Results from two separate experiments. In both experiments, tp53+/-;fgf24+/- fish were incrossed and grown until 2 or 4 mpf. Euthanized fish were fixed and phenotyped for sex, and gonads were dissected and counted. Fish were then genotyped for both tp53 and fgf24. Since all genotypes were reared together, we would expect similar sex ratios. Unlike tp53 single mutants, however, tp53;fgf24 double mutants are all phenotypic males usually with zero or one gonad, similar to fgf24 single mutants. (TIF) [file pgen.1006993.s009.tif]
