# Supplementary material for: Fibroblast growth factor signaling is required for early somatic gonad development in zebrafish
Source: PLoS Genet. 2017 Sep 5;13(9):e1006993. doi: 10.1371/journal.pgen.1006993 (PMC5600409; doi:10.1371/journal.pgen.1006993)
Supplement: S1 Methods — (DOCX) [file pgen.1006993.s010.docx]

**Genotyping primers**

|  | Forward (5’-3’) | Reverse (5’-3’) |
| --- | --- | --- |
| *fgf24^hx118^* | TGGTCTTTTGTATTTTGCAGCTCT | CGAACGTGACTCCCGAAA |
| *tp53^zdf1^* | ACTACATGTGCAATAGCAGCTGC | CTCCTGAGTCTCCAGAGTGATGA |
| *fgf24^uc47^* | CAGCTCTACAGCCGAACCACCGGCA | AGACTCCCGTACCGTACTTGCCCCC |
| *Tg(ziwi:EGFP)^uc02^* | CTCAGAGGTTTAGAACTACGTGAGG | AGATGAACTTCAGGGTCAGCTTGC |
| *Tg(gsdf:mCherry)^uc46^* | CAGGACGGCGAGTTCATCTA | CTGCTTGATCTCGCCCTTCA |

***in situ* hybridization probe synthesis**

|  | Forward (5’-3’) | Reverse (5’-3’) | Plasmid | Linearized with | RNA polymerase |
| --- | --- | --- | --- | --- | --- |
| *fgf24* | ATACACAGTCCTGTACCATC | GTATTGGGGTTGGGTTTTA | pGEM-T Easy | NcoI | Sp6 |
| *etv4* | Munchberg et al., 1999 |  | pCR4 Topo | NotI | T7 |
| *gata4* | Reifers et al., 2000 |  | pCRII Topo | XbaI | T7 |
| *cyp19a1a* | Siegfried & Nusslein-Volhard, 2008 |  |  | SpeI | T7 |
| *amh* | Siegfried & Nusslein-Volhard, 2008 |  |  | SpeI | T7 |
| *nr5a1a* | GACTTCCTCTGCTACAAACACC | AAGTTGCAGACGCCTTTACC | pGEM-T Easy | XmnI | Sp6 |
| *wt1a* | ATGGGTTCTGATGTTCGTGACC  Bollig et al, 2006 | ACATCCTGAAGGCCAGTGAAGAC | pGEM-T Easy | SpeI | T7 |
| *gsdf* | CTGCTGCGCCTCGTCCTGCT | ACGGAGGGCTCATGGCTGCG | pCRII Topo | NcoI | T7 |

**Antibodies**

| Antigen | Source | Catalog # | Dilution | Notes |
| --- | --- | --- | --- | --- |
| Vasa | Knaut et al., 2000 | -- | 1:2500 | Works well after proteinase K treatment, ISH |
| pErk1/2 | Sigma | M8159 | 1:200 | Does not work after ISH |
| Cleaved caspase 3 | Cell Signaling | 9661 | 1:400 |  |
| pHH3 (Ser10) | Millipore Sigma | 05-598 | 1:250 |  |
| Laminin | Sigma | L9393 | 1:300 | Does not work after proteinase K, ISH |
| Beta catenin | Sigma | C7207 | 1:500 |  |
| E-cadherin | BD Biosciences | 610181 | 1:100 |  |
| N-cadherin | GeneTex | GTX125885 | 1:200 | Sub-optimal after ISH |
| Tjp-1/  ZO-1 | Life Technologies | 339100 | 1:100 | Does not work after proteinase K, ISH |
| mouse  (-HRP conj) | ThermoFisher | G-21040 | 1:500 |  |
